# Neoantigens: promising targets for cancer therapy

**DOI:** 10.1038/s41392-022-01270-x

**Published:** 2023-01-06

**Authors:** Na Xie, Guobo Shen, Wei Gao, Zhao Huang, Canhua Huang, Li Fu

**Affiliations:** 1 State Key Laboratory of Biotherapy and Cancer Center, West China Hospital, and West China School of Basic Medical Sciences and Forensic Medicine, Sichuan University, and Collaborative Innovation Center for Biotherapy, Chengdu, 610041 China; 2 Clinical Genetics Laboratory, Affiliated Hospital & Clinical Medical College of Chengdu University, Chengdu, 610081 China; 3 Guangdong Provincial Key Laboratory of Regional Immunity and Diseases, Department of Pharmacology and International Cancer Center, Shenzhen University Health Science Center, Shenzhen, 518060 Guangdong, People’s Republic of China

**Keywords:** Predictive markers, Tumour immunology, Cancer, Predictive medicine, Biomaterials

## Abstract

Recent advances in neoantigen research have accelerated the development and regulatory approval of tumor immunotherapies, including cancer vaccines, adoptive cell therapy and antibody-based therapies, especially for solid tumors. Neoantigens are newly formed antigens generated by tumor cells as a result of various tumor-specific alterations, such as genomic mutation, dysregulated RNA splicing, disordered post-translational modification, and integrated viral open reading frames. Neoantigens are recognized as non-self and trigger an immune response that is not subject to central and peripheral tolerance. The quick identification and prediction of tumor-specific neoantigens have been made possible by the advanced development of next-generation sequencing and bioinformatic technologies. Compared to tumor-associated antigens, the highly immunogenic and tumor-specific neoantigens provide emerging targets for personalized cancer immunotherapies, and serve as prospective predictors for tumor survival prognosis and immune checkpoint blockade responses. The development of cancer therapies will be aided by understanding the mechanism underlying neoantigen-induced anti-tumor immune response and by streamlining the process of neoantigen-based immunotherapies. This review provides an overview on the identification and characterization of neoantigens and outlines the clinical applications of prospective immunotherapeutic strategies based on neoantigens. We also explore their current status, inherent challenges, and clinical translation potential.

## Introduction

Accumulating genetic alterations in cancers result in the production of tumor-specific antigens (TSAs) or neoantigens, which can be presented by major histocompatibility (MHC) molecules of tumor cells.^1–6^ These tumor-specific peptide-MHC (pMHC) complexes are recognized by T cells and trigger an anti-cancer immune response in patients. However, it has been discovered that cancer cells have evolved resistance to anti-cancer immunity.^7^ These immune escape mechanisms can be reversed by cancer immunotherapies, including the use of tumor vaccines to improve antigen presentation, the increase of anti-tumor T cells via adoptive transferring of tumor-infiltrating lymphocytes (TILs) and T cell receptor (TCR)-transduced T cells, restoring the effector capacity of CD8^+^ T cells by immune checkpoint blockades (ICBs), increasing the immune recognition of tumors with bispecific antibodies (bsAbs) and chimeric antigen receptor (CAR)-transduced T cells, and modulating the tumor immune microenvironment.^8–19^ A variety of clinical studies examined the efficacy of immunotherapies targeting tumor-associated antigens (TAAs), like vaccines against ERBB2, MUC1, and hTERT. TAAs exhibit abnormal expression in malignancies or are only produced during specific stages of differentiation, whereas their expression in normal tissues is extremely limited. The prevalence of TAAs among cancer patients makes them public targets for off-the-shelf immunotherapies. However, as TAAs are non-mutated self-antigens, central T cell tolerance may contribute to the largely poor T cell responses observed in clinical trials.^20,21^ Nonetheless, the widespread use of tumor immunotherapies has been hindered by a shortage of targetable antigens in various cancers.^22^

Neoantigens are self-antigens generated by tumor cells because of genomic mutations. Besides, neoantigens can also derive from unique proteins or peptides produced by dysregulated RNA splicing and disordered post-translational protein modification in non-virus-associated malignancies. For cancers with a viral infection, such as HPV-positive cervical cancer and EBV-associated nasopharyngeal carcinoma, neoantigens can also be created by virally encoded open reading frames (ORFs).^22–24^ Compared with other types of tumor antigens, such as cancer-testis antigens (CTAs) and TAAs, neoantigens offer a distinct advantage in their unique tumor-specific and absence in normal tissues, presenting ideal targets for effectively personalized treatment of tumors (Table 1).^25,26^ Notably, T cells specialized for neoantigens can bypass negative selection effects in the thymus due to the highly antigenic neoantigens acquired through somatic tumor mutations. Increasing the pool of neoantigen-specific T cells due to this ability to avoid T cell central tolerance makes it possible to enhance tumor-specific immune responses.^27–29^ Furthermore, the capacity of immunotherapy-enhanced neoantigen-specific T cell responses in enduring and giving post-treatment immunological memory offers hope for long-term protection against disease recurrence.^30^

**Table 1:** The characteristics of TAAs and neoantigens

	Tumor antigens	Specifical expression	Central tolerance	Autoimmune toxicities
Tumor-associated antigens (TAAs)	Overexpressed proteins, lineage-specific differentiation markers	Tumors	The central and peripheral tolerance	Colitis, renal impairment, severe hepatitis, rapid respiratory failure, treatment-related death
	Cancer germline antigens (CGAs)	Tumors, testes, fetal ovaries, trophoblasts	The central and peripheral tolerance	Colitis, renal impairment, severe hepatitis, rapid respiratory failure, treatment-related death
Tumor-specific-antigens (TSAs)	Oncoviral antigens	Virus-associated tumors	Non-central tolerance	Less
	Neoantigens	Tumors	Non-central tolerance	Less

**Fig. 1:**
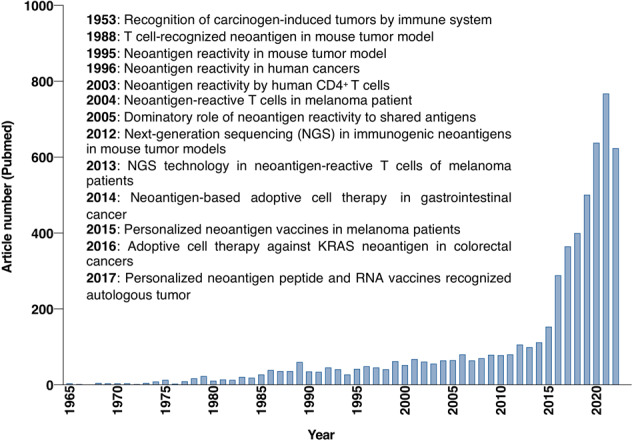
Historical overview of tumor-specific neoantigens. Based on keyword searches in the PubMed database using the terms "neoantigen" or "neoepitope", the number of articles from 1965 to 2022 is displayed in the column chart

Currently, advanced techniques, including tumor gene sequencing, neoantigen discovery, and neoantigen-based immune therapeutic product preparation, play a significant role in the development of personalized cancer vaccines (PCVs) and adoptive cell therapy (ACT).^19^ Next-generation sequencing (NGS) has permitted the fast and cost-effective detection of tumor-specific mutations in individual patients. In addition, the development of algorithms for predicting MHC molecules-binding epitopes has made it possible to identify possibly immunogenic neoepitopes.^31^ These technological developments have enabled the production of personalized immunotherapy, specifically targeting tumors in individual patients (Fig. 1). However, some limitations such as the costs and time in the process of personalized immunotherapeutic products, and the ideal platform for neoantigen identification, need further improvement. With the continuous development and wide cross-integration of biotechnology, immunology, materials science, chemistry, and artificial intelligence, additional neoantigens will be identified and employed in tumor immunotherapy.^13,32^

Herein, we provide a comprehensive summary of the source and biological function of neoantigens, potential neoantigen prediction tools, and clinical applications of neoantigen-based immunotherapy strategies. Moreover, we also discuss the opportunities and limitations associated with the clinical application of immunotherapies based on neoantigens and propose some potential solutions.

## The source of neoantigens

**Fig. 2:**
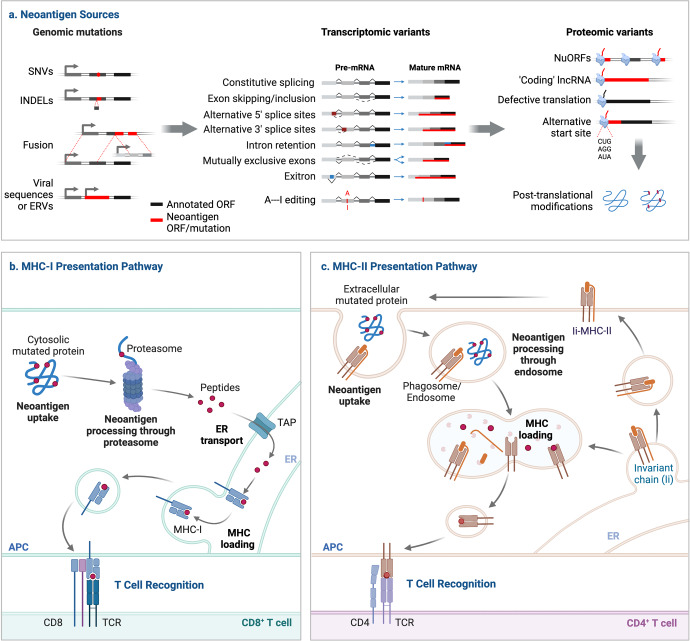
Overview of the neoantigen production and presentation. Neoantigens can develop at the genomic level through SNVs, base INDELs and gene fusions, at the transcriptomic level through alternative splicing, polyadenylation (pA), RNA editing and allegedly non-coding regions, and at the proteomic level through dysregulated translation and PTMs. The integrated viral ORF is another source of neoantigens for cancers linked to viruses. The mutant peptides created by the proteasome-mediated breakdown of endogenous proteins are subsequently transported to the endoplasmic reticulum (ER) via transporters associated with antigen processing (TAP), where they may be loaded onto MHC-I. MHC-II dimers are assembled and bound to the invariant chain (Ii) in the ER. The Ii-MHC-II complex can be directly transported or sometimes indirectly internalized from the cell surface to the MHC-II compartment (MIIC), where the degradation of Ii by a series of endosomal proteases releases the MHC-II for binding a specific peptide derived from a mutant protein broken down in the endosomal pathway. These pMHC complexes will then traffic to the cell surface where they are recognized by T cells

Neoantigens are identified as foreign proteins that are absent in normal tissues, but can arise from tumors through various mechanisms, such as genomic mutation, aberrant transcriptomic variants, post-translational modifications (PTMs), and viral ORFs (Fig. 2).^27,33^

### Genomic variants

Somatic genomic alterations, including single-nucleotide variants (SNVs), base insertions and deletions (INDELs) and gene fusions, are the main factors that promote the production of tumor neoantigens.^8,34–37^

### SNVs

SNVs are the most prevalent type of mutation at the genomic level in tumor cells; they can yield variant peptides distinct from wild-type proteins that are presented by MHC-I as specific antigens.^19,38^ Up to hundreds of non-synonymous somatic mutations per cancer patient have been recorded, resulting in an average of 150 potential neoantigenic peptides per individual. For example, a total of 231 non-synonymous SNVs, 13 gene fusions and 21 INDELs have been identified in Ph-negative myeloproliferative neoplasms (MPNs).^39^ Using The Cancer Genome Atlas (TCGA) database, 933,954 expressed neoantigens in 20 solid tumors have been characterized, which originates from 893,960 somatic mutations with a varied median frequency of neoantigens across cancers. Only 24 of these neoantigens, including those arising from mutations in driver genes like PIK3CA, RAS, and BRAF, are shared by at least 5% of patients with the same or distinct cancers.^38,40^ Notably, relapsed populations may have greater tumor mutation burden (TMB) and more novel potential neoantigens than newly diagnosed patients. In patients with multiple myeloma, only two potential neoantigens, UBR4 and PRKDC, were detected in both relapsed and newly diagnosed patients.^41^ Therefore, the SNV neoantigens landscape is highly variable between different cancer types and different stages of the same cancer type (Table 2).

**Table 2:** Advantages, disadvantages and relevant cancers for each class of neoantigens

Neoantigen classification	Sources	Advantages	Disadvantages	Relevant cancer
Genomic variants	Single-nucleotide variants (SNVs)	Simple prediction; relatively high burden	Similar to self-antigen; rarely shared between patients	Melanoma, glioblastoma, lung cancer (adeno and squamous), bladder cancer
	Insertions and deletion (INDEL) frameshift	More potential targets per mutation; more dissimilar from self-antigen; more immunogenic	Relatively low burden	MSI-H tumors, renal cell carcinomas (clear cell, papillary, and chromophobe)
	Fusion genes	More dissimilar from self-antigen; shared targets between tumors; more potential targets per mutation; more immunogenic	Relatively low burden	Acute myelocytic leukemia, acute lymphocytic leukemia, chronic myeloid leukemia, sarcomas
	Chromosomal rearrangements	High immunogenicity	Less well studied	Malignant pleural mesothelioma
Transcriptomic variants	RNA splicing	A large number of predicted targets; More dissimilar from self-antigen	Fewer tools available; not well validated in pre-clinical models; current tools do not account for nonsense mediated decay (NMD)	Acute myelocytic leukemia, chronic myelomonocytic leukemia, chronic lymphocytic leukemia, myelodysplastic syndrome
	Polyadenylation (pA) and RNA editing	Easy prediction	Less well studied	Chronic lymphocytic leukemia
	Allegedly non-coding regions	Relatively high burden; more potential targets	Less well studied; fewer tools available	Acute lymphoblastic leukemias, lung cancers
Proteomic variants	Post-translational modifications (PTMs)	Shared between patients	Less well studied	leukemia, renal cancer, non-small cell lung cancer
	Proteasome processing	High specificity	Less well studied; fewer tools available	Acute myeloid leukemia
	T cell epitopes associated with impaired peptide processing (TEIPP)	TEIPP-specific T cells can escape thymic selection	Less well studied; limited in HLA-I low or TAP-deficient tumors	Lung cancer
Viral-derived neoantigens	Viral open reading frames	High immunogenicity; more dissimilar from self-antigen; shared between patients; without apparent toxicity to normal tissues.	Limited in specific tumors	Hepatocellular carcinoma, Merkel cell carcinoma, nasopharyngeal carcinoma, head and neck cancer, cervical cancer, anal cancers

SNVs can also arise in mitochondrial DNA (mtDNA), which is found in most cancer cells and correlates with alterations in tumor metabolic profiles and cancer cell metastatic capacity.^42–45^ Despite the fact that the compact normal human mitochondrial genome, a 16,569 base-pair circular DNA, encodes only 13 protein subunits of the electron transport chain, it may account for around 30% of total mRNA transcripts in certain organs.^46^ mtDNA has a 10- to 20-fold greater mutation rate than nuclear DNA.^47,48^ Both mouse and human immune systems were able to recognize and respond to mtDNA single nucleotide polymorphisms (SNPs)-derived peptides, suggesting that individual SNPs in mtDNA are adequate to generate immunogenic neoantigens. Thus, non-synonymous SNPs in mtDNA may yield a substantial quantity of mutant peptides, offering an additional source of neoantigens.^49–51^

### INDELs

INDEL mutations are mainly caused by the insertion or deletion of base pairs in the genome, which frequently lead to non-synonymous novel ORFs, also known as frameshift mutations.^27,52^ Frameshift INDELs can generate more types of neoantigens with increased MHC-I binding affinity, suggesting a higher immunogenic mutation type compared to SNVs (Table 2).^53,54^ Especially in renal cell carcinoma with a medium-range mutational burden, about 16% of predicted neopeptides are derived from frameshift INDELs, whereas 21% of T cell-recognized neoepitopes are arising from frameshift INDELs, indicating that frameshift-derived neoepitopes have a greater immunogenic potential.^53,55^

Similar to SNV neoantigens, INDEL neoantigens are more prevalent in cancers with microsatellite instability-high (MSI-H) due to the lack of DNA mismatch repair (MMR) mechanisms.^27,56–58^ As the evolution of MMR-deficient cancers is mainly triggered by mutations that inactivate tumor suppressor genes (TSGs) containing coding microsatellites, frameshift peptide neoantigens are more frequently shared among MMR-deficient cancers (e.g., endometrial, colorectal, and gastric) than missense mutation-derived neoantigens.^59–64^ Frameshift INDEL neoantigen burden has a strong correlation with immunological response.^38^ MSI colorectal cancers with frameshift mutations have a larger proportion of TILs than other colorectal cancers.^64–66^ Similarly, shared immunogenic frameshift peptide neoantigens can be produced as a result of recurrent frameshift mutations, offering excellent candidates for immunotherapy against MSI cancers.^59,61–64^ The combination of four frameshift peptide neoantigens dramatically boosts neoantigens-specific adaptive immunity, decreases intestinal tumor burden, and prolongs the overall survival in the VCMsh2-driven intestinal cancer mouse model, which can be further strengthened by naproxen.^67,68^ According to a clinical phase I/IIa trial, the frameshift peptide neoantigenic vaccine is well tolerated systemically and triggers immune responses regularly, representing a promising new strategy for the treatment and even prevention of MMR-deficient malignancy.^60^ These findings showed that an off-the-shelf vaccine is feasible for treating and preventing cancers with frameshift mutations and neoantigenic peptides because of MSI.^69^

The frameshift INDEL neoantigen burden is also a novel biomarker for ICB response.^27,38,70–72^ INDEL frameshift mutations are supposed to produce more immunogenic neoantigens, hence improving response to ICBs. When frameshift mutations are present, the progression-free survival of patients receiving ICBs is significantly prolonged. Further evidence that frameshift mutations may play a predictive role in ICB response comes from the considerable discrepancies in overall response rates and disease control rates observed in non-small cell lung cancer (NSCLC) patients with frameshift mutations.^73^ In addition, ICBs can also strengthen the immune response to frameshift neoantigens. The frameshift mutation in CALR elicits both CD4^+^ and CD8^+^ T cell responses, which are inhibited by the expression of PD-1 or CTLA4. Importantly, blocking PD-1 and CLTA4 ex vivo and PD-1 in vivo with pembrolizumab restores frameshift neoantigen-specific T cell immunity in myeloproliferative neoplasms.^39,74^

### Gene fusions

Gene fusion is another important type of mutation in tumors that may provide many neoantigens, which can be generated by mesenchymal deletion, chromosomal translocation or chromosomal inversion.^28,75–77^ Studies have shown that polypeptides derived from the different fusion regions of the proteins can be recognized by the patient’s own T cells, such as the BCR-ABL fusion protein produced by the translocation between chromosomes 9 and 22 in chronic myeloid leukemia (CML) patients and SYT-SSX1 fusion proteins produced by X and 18 chromosomal translocations in synovial sarcoma patients. Even in some tumors with low TMB and limited immune infiltration, neoantigens generated by gene fusion are still able to activate cytotoxic T cells.^21,78,79^ In a comprehensive study of fusion neoantigens in tumors, analyses of three datasets from the TCGA database found that fusion mutations could generate more novel ORFs, yielding 6-fold neoantigens and 11-fold specific candidate neoantigens more than SNVs and INDELs. The fusion neoantigens are more likely to induce stronger immune response than the neoantigens produced by SNVs and INDELs, and the neoantigen produced by frameshift fusion has better immunogenicity than the in-frame fusion neoantigen (Table 2). Similar to the candidate neoantigen burden of SNVs and INDELs, fusion neoantigen burden was closely related to fusion mutation burden, especially in microsatellite stable tumors with higher fusion mutation burden.^19,80^ An expanded study of 30 different tumor types revealed that 24% of fusion protein-expressing cancers contained neoepitopes resulting from the fusion, and these neoantigens were predicted to bind to patient-specific MHC-I.^78,81^ It is worth noting that the repetition rate of fusion neoantigens between different patients is extremely low. According to statistics, only 5.8% of fusion neoantigens in the TCGA database repeat between patients, and these neoantigens usually have very low immunogenic potential.^80^ In addition, malignancies with greater immune-depleted microenvironments or human leukocyte antigen (HLA) loss exhibited fusion neoantigens more frequently. In melanomas treated with anti-PD-1 therapy, the removal of tumor cells carrying fusion-derived neoantigens demonstrated a negative immune surveillance selective pressure on these neoantigens.^78,82^ According to FACETS analysis of the TCGA exome data, 18.4% of cases had a loss of heterozygosity (LOH) in the HLA, which increased the possibility that a fusion neoantigen would be present.^78,83^ These results demonstrate the significance of gene fusions as a source of tumor-specific neoantigens.^78,81^

Gene fusion-derived neoantigens can elicit specific immune responses against tumors.^84,85^ The fusion neoantigens, such as BCR-ABL, SYT-SSX1/SSX2, PAX3-FOXO1, TPM3/TPM4-ALK, and EWS-FLI1, showed immunogenic potential, providing the possible targets for immunotherapy to treat tumors.^86,87^ CBFB-MYH11 fusion neoantigen is distributed on acute myeloid leukemia (AML) cells, which activates T cells and induces specific killing against AML, a cancer with low mutation frequency.^88–91^ Two neoantigens, SS393 (GYDQIMPKK) and SS391 (PYGYDQIMPK), are derived from the SYT-SSX fusion neoantigen that is common in synovial sarcoma. These neoantigen peptides successfully induced synovial sarcoma-specific cytotoxic T lymphocytes (CTLs) that specifically killed HLA-A24-positive synovial sarcoma cells containing the SYT-SSX neoantigen as well as the target cells pulsed with these peptides.^92–94^ A study on head and neck squamous cell carcinomas (HNSCC) found that the tumor’s immune response to anti-PD-1 therapy was mediated by neoantigens generated by DEK-AFF2 fusion. A DEK-AFF2-derived peptide (DKESEEEVS) enhanced T cell activation depending on MHC class when delivered to autologous peripheral blood mononuclear cells (PBMCs).^78^ A comprehensive study of 33 tumor types found that various common recurrent fusion neoantigens, including TMPRSS2-ERG, MYB-NFIB, FGFR3-TACC3, EML4-ALK and CCDC6-RET.^95^ TMPRSS2-ERG is the most common recurrent gene fusion that occurred in 38.2% of prostate cancer patients. Several high-affinity HLA-restricted epitopes were identified from the recurrent TMPRSS2-ERG type VI fusion, which could bind to HLA-A*02:01 in vitro and were recognized by CD8^+^ T cells.^96^ The fusion of the proto-oncogene MYB with the transcription factor NFIB serves as a biomarker for adenoid cystic carcinoma, which occurs in 60% of cases. Three MYB-NFIB-derived peptides (QFIDSSWYL, SLASPLQPT and SLASPLQSWYL) and one NFIB-MYB-derived peptide (MMYSPICLTQT) can bind to HLA-A*02:01 to activate the immune system.^78,97^ The EML4-ALK fusion gene is predominantly found in young, rarely/never smoker NSCLC patients, and ~5% of NSCLC patients have this fusion mutation. The use of EML4-ALK-derived peptides can stimulate specific CTL responses and have the potential to treat EML4-ALK-positive NSCLC.^98^ Therefore, the neoantigens generated by fusion mutations greatly increase the capacity of the tumor-specific neoantigen repertoire, providing more potential targets or predictors for cancer immunotherapies.^8,78,81,88,96,99–101^

### Structural variants

Structural variant (SVs) is one of the most frequent forms of driver mutations in tumors, which can result in alterations in genome structure and then change the expression or function of genes to promote malignant transformation. SVs generally refer to genetic variants that are larger than 50 base pairs, such as insertions, deletions, inversions, translocations, duplications/amplifications, and chromosomal additions and deletions, as well as chromosomal rearrangements.^5,102–109^ Among them, chromosomal rearrangement is the most complex (such as chromothripsis and chromoplexy), which is a prominent feature of tumors and plays a crucial role in the occurrence and immune recognition of various malignant tumors.^110–112^ Chromosomal rearrangements are not easily detected by traditional DNA sequencing techniques but can be screened by WES methods like mate pair sequencing (MPseq). Potential neoantigens generated by chromosomal rearrangements have been identified by a combination of MPseq and RNA sequencing (RNA-seq) in malignant pleural mesothelioma (MPM) patients. Rearrangement-related neoantigens may generate MPM-specific immune responses in a manner similar to frameshift INDEL neoantigens. Specifically, neoantigens produced by SVs are predicted to be presented by tumors on MHC proteins, which are closely related to clonal expansion of TILs, and effector T cells against these neoantigens are found in the circulation of cancer patients.^110,113^ Therefore, SV-derived neoantigens may also serve as valuable targets for anti-tumor immunotherapy.

### Transcriptomic variants

Post-transcriptionally events offer the potential of a broadened neoantigen space. Alternative processing of mRNA, including alternative splicing events, polyadenylation (pA), RNA editing and allegedly non-coding regions, contributes to the diversity of tumor-specific neoantigens.^19,81,114–116^

### Transcript alternative splicing

The abnormal alternative mRNA splicing is another potential source of tumor-specific neoantigens.^22,117^ RNA splicing process the premature mRNA into mature RNA with high efficiency and fidelity in normal cells. However, it may be induced by mutations in RNA cis-regulatory elements, trans-acting regulators or the core spliceosome.^117–119^ The highly aberrant splicing events in tumors expand the scope of tumor-specific neoantigens, especially in tumors with low rates of copy number variation and somatic mutations.^23,117,120^

#### Cis-acting mutations

Mutation at cis-acting elements generates potential neoantigens through altered splicing, including alternative 5’ and 3’ splice site determination, intron retention, exon skipping and mutually exclusive exons.^23,33,121,122^ Intron retention is more prevalent in nearly all cancers compared with normal control tissues, even in the absence of mutations in genes encoding splicing factors. Normally, intron retention transcripts will be degraded by nonsense-mediated RNA decay (NMD). Neoantigens can still be generated from intron retention during their pioneer round of translation before being subjected to NMD.^33,117,123,124^ Numerous exon-exon junctions that are unique in tumors have been identified through extensive study of the TCGA, most of which can express neoantigens.^117,125,126^ The production of neoantigens from skipped exons, also known as “neojunctions”, occurs more frequently and is more likely to be shared among patients than those generated from SNV mutations.^29,117,127,128^ A recent study has identified exitron splicing, a non-canonical splicing mechanism, as a new source of tumor neoantigens. Exitrons are exon-embedded cryptic introns distinguished from conventional introns in that they have both splicing (intron) and protein-coding (exon) potential while lacking stop codons or premature termination codons. Because tumor-specific exitron-spliced transcripts are far more likely to escape NMD than intron retentions, their overall expression is higher than retained introns. Accordingly, exitrons splicing creates more validated neoantigens with higher immunogenicity in malignancies with low TMB.^128,129^

#### Trans-acting alterations in splicing factors

Trans-acting alterations, in which a somatic mutation in a splicing factor results in an altered splicing variant, induce the production of neoantigens throughout the genome.^130^ In hematological malignancies, common mutations in spliceosomal components, including SRSF2, SF3B1, and U2AF1/2, raise the expression of splice variant mRNAs, resulting in the translation of TSAs and neoantigens. In addition to hematological tumors, a recent reassessment of pan-cancer data in the TCGA database has shown that the somatic alterations of splicing factors, including SF3B1, U2AF1, SRSF2 and Zinc Finger CCCH-Type, ZRSR2, U2AF2, SF1, PRPF8, and SF3A1, leading to the production of splicing variant-derived neoantigens across the genome in solid tumors.^22,27,117,119,131–138^ Moreover, epigenetic alterations and PTMs of splicing factors might promote global splicing dysregulation.^139^ Neoantigens derived from splicing variants due to mutation and dysregulated expression of splicing factors have facilitated the development of novel therapeutics for tumors. For example, mutated SF3B1 (a splicing factor in the spliceosome) in uveal melanoma generates tumor-specific neoantigens that activate specific CD8^+^ T cells to kill tumor cells.^140^

#### Nonsense-mediated RNA decay (NMD)

Another important determinator for tumor-specific splicing variants is NMD, a highly conserved RNA turnover mechanism that preferentially destroys RNAs carrying premature translation termination codons. In cells with normal NMD function, a pioneer round of translation is required for initiating the NMD-mediated degradation of aberrant transcripts, which can lead to the production of small amounts of neoantigens.^23,123,141,142^ Moreover, the NMD regulatory mechanism is frequently impaired in tumor cells, enabling aberrant transcripts to avoid degradation and potentially produce large amounts of neoantigens. For example, mutations in the highly conserved core NMD factor UPF1 are prevalent in pancreatic squamous cell carcinoma and lung adenocarcinoma, increasing the frequency of aberrant transcripts and neoantigen production.^143–145^ A recent study demonstrated that NMD regulates the mutational profile of malignancies by preferentially suppressing the expression of TSGs rather than oncogenes. Further evidence for the beneficial effect of NMD on tumors comes from the observation that NMD frequently degrades mRNA encoding immunogenic neoantigen peptides. Accordingly, NMD inhibitory therapy may be beneficial in the treatment of a variety of cancers, including those capable of producing large numbers of mutated neoantigens.^146,147^

Altogether, these studies highlight that alternative splicing of transcripts could promote the production of neoantigens. Even though the application of splicing variant neoantigens in personalized therapies has not yet been thoroughly investigated, screening new alternative splicing-based neoantigens as immunotherapeutic targets will benefit tumor patients.^22,23,27^

### Polyadenylation (pA) and RNA editing

Similar to RNA splicing, polyadenylation (pA) and RNA editing can alter the proteomic profile of tumor cells, thereby increasing the pool of potential immunotherapeutic targets.^81,148^

Polyadenylation plays a critical role in the processing and maturation of most eukaryotic mRNAs, primarily by cleaving and adding a poly(A) tail at the 3’ end. Most alternative polyadenylation (APA) events occur in the 3ʹ untranslated region (UTR) of mRNA. APA can significantly affect post-transcriptional gene regulation in several aspects, including transcript stability, translation, cellular localization, and nuclear export.^149–154^ Nonetheless, some APA events occur in the intronic region upstream of the last exon, which is called intronic polyadenylation (IPA).^155,156^ IPA can result in the production of truncated or non-coding transcripts that have the potential to generate tumor-specific immunotherapeutic targets. A recent study used 3’ end sequencing technology to analyze normal and malignant B cells of 59 patients with chronic lymphocytic leukemia (CLL), the study found that IPA-induced mRNA and protein truncations are prevalent in CLL cells, mainly involving TSGs such as DICER and FOXN3, and even some oncogenes such as CARD11, MGA, and CHST11.^157^ It is worth noting that 72% of the 190 TSGs found in hematological tumors are only truncated in solid tumors.^158^ In tumors, when a specific IPA event occurs in the coding region, genes upstream of the new pA site and downstream of the closest 5ʹ splice site are translated, creating neoantigens that can be presented by MHC and recognized by the immune system.^81^ By comparing RNA-seq data between tumor and normal tissue samples from various cancers, more neoantigens created by IPA might be identified, providing prospective targets for cancer immunotherapy.

RNA editing is an important pre-mRNA processing method that can induce non-synonymous substitutions by altering specific nucleotides in the RNA sequence, resulting in the production of new proteins.^38,159^ Similar to splicing and polyadenylation, RNA editing events frequently occur in a variety of tumors.^160–164^ Adenosine-to-inosine (A-to-I) editing is the most prevalent type of RNA editing in mammals, and millions of such sites have been found in human genes. Protein peptides produced by A-to-I editing can be presented by MHC-I molecules, which further induce the activation of specific CD8^+^ T cells, suggesting that these novel peptides are immunogenic and can activate the immune system.^165–169^ Nevertheless, it must be emphasized that these peptides are not necessarily tumor-specific, as RNA editing can also occur in normal tissues. Therefore, more in-depth research and advanced prediction methods are needed to identify tumor-specific RNA editing protein products for immunotherapy.^81,170^

### Allegedly non-coding regions

Given that 99% of tumor-specific mutations occur in non-coding regions of genes, and exonic regions account for only 2% of the entire human genome, the screening for neoantigens only derived from mutations in exonic regions is limited. Recent studies showed that many regions previously defined as non-coding are now found to have coding functions. Therefore, by studying these newly defined genes with coding capacity, researchers have discovered many novel antigenic peptides that can be presented by MHC-I, and some antigens have been confirmed as target for TIL immunotherapy.^171–173^ These MHC-I-associated peptides (MAPs) derived from genes at non-coding regions expand the range of CD8^+^ T cell immune surveillance from 2% (the proportion of the human genome in exons) to 75%.^52,172,174,175^ According to a proteogenomic profiling of non-canonical proteins, 60% of non-canonical proteins are encoded by genes that were considered to be located at non-coding regions previously. More recently, using the mass spectrometry (MS) methods, many sorts of non-coding regions have been identified to produce large amounts of aberrantly expressed tumor-specific antigens, the bulk of which originate from epigenetic modifications in atypical translation events rather than mutations.^176^ These aberrantly expressed tumor-specific neoantigens are more prevalent than neoantigens created by mutations in coding areas and can be shared between tumor patients.^27,172,177^ Numerous such cryptic peptides were found in tumor immunopeptidomes using Peptide-PRISM. The presentation of cryptic peptides is HLA-I allele dependent, with HLA-A*03 and HLA-A*11 showing the largest proportion of cryptic peptides.^178^ Critically, cryptic proteins create MHC-I peptides five times more efficiently per translation event than canonical proteins do, due to their more predicted disordered residues and lower stability.^179^ No studies have reported that MHC-II-associated neoantigens generated from non-coding regions may activate CD4^+^ T cells. Compared with other mutations at the genome and transcriptome level mentioned in this review, neoantigens derived from the translation of non-coding regions are rarely clearly understood. Therefore, it is urgent to develop fast and efficient computational algorithms to screen these potential neoantigens and to verify their feasibility for immunotherapy.^38^

### Proteomic variants

Dysregulated translation that is a characteristic of carcinogenesis offers an important new source of tumor-specific neoantigens.^180^ In addition, the proteomic variants also come from the aberrant function of PTMs, proteasome processing, and transporter associated with antigen processing (TAP).^181–184^

Neoantigen presentation by MHC molecules to T cells can maintain specific PTMs.^181,182^ Aberrant PTMs, including glycosylation, O-linked β-N-acetylglucosamine (O-GlcNAc) and phosphorylation, can create neoantigenic peptides presented by MHC complexes in tumors.^185^ For example, the neoantigen arising from post-translationally modified MUC1 was presented by MHC-I and exclusively recognized by a glycoform-specific TCR.^182^ Moreover, an unusually large proportion of mutations may enhance the formation of novel N-glycosylation sites, resulting in generation of neoantigens.^186^ Five O-GlcNAc modified peptides in leukemia were found to induce multifunctional memory T cell responses in healthy donors. Neoantigens derived from O-GlcNAc modified proteins explain why leukemias are highly immunogenic despite having a low mutational load, thereby offering prospective therapeutic targets.^187^ Dysregulated phosphorylation can generate neoantigens by promoting the binding of epitopes to MHC molecules or by altering the antigenic features of presented epitopes.^188^ The cancer-associated phosphopeptides derived from insulin receptor substrate 2 (pIRS2) and breast cancer antiestrogen resistance 3 (BCAR3) were immunogenic in vivo in mice, and in vitro in normal human donors.^189,190^ Several T cell lines have demonstrated a specifically recognition of the post-translationally modified peptide but not the unmodified peptide, indicating that the aberrant PTMs results in a different neoantigen and cognate TCR.^182,187^ Notably, immunogenic peptides derived from dysregulated PTMs in cancer cells constitute an unexplored class of tumor-specific neoantigens that could serve as off-the-shelf targets for cancer immunotherapy. PTMs can also be employed to produce unique neoantigens to improve the immune recognition of cancer cells. Covalent KRAS-G12C inhibitors, like ARS1620, result in covalently modified peptides, which can be presented on MHC-I to elicit T cell response. These tumor-specific PTMs, which involve the covalent drug-mediated alkylation of mutant cysteine residues on oncoproteins, provide a novel source of neoantigens that can be readily targeted by immunotherapies.^191,192^

Another repertoire of neoantigenic epitopes is derived from impaired proteasome processing or TAP complexes. The proteasome processes proteins and converts them into peptides, which is particularly critical for transforming proteins into MHC-restricted epitopes. The oxidants like peroxynitrite generated by myeloid cells in tumor microenvironment (TME) inhibit the activity of proteasome, thereby decreasing the production of MHC-I peptides.^193,194^ Protein splicing significantly increases the proteome complexity of malignancies, which alters the hierarchy of antigenic epitopes.^195,196^ Studies have also revealed that the proteasome can produce novel immunoreactive spliced epitopes (splicetopes) by fusing with peptide fragments excised by reverse proteolysis during proteasome-catalyzed peptide splicing (PCPS), which differ from the original substrate protein sequence.^196–198^ According to preliminary statistical analysis, the proteasome is responsible for splicing around one-third of MHC-I-related immune peptides.^199^

There is evidence that neoantigens involving the linkage of existing individual peptides can activate CD4^+^ T cells in type 1 diabetes (T1D), indicating that proteomic variants processes may generate MHC-II-associated neoantigens.^200^ Several studies have reported that the spliced peptides produced by the proteasome are able to activate CD8^+^ T cells.^198,201,202^ Splicetope-specific CD8^+^ T cells from TILs isolated from human AML patients inhibited the growth of their corresponding tumor cells in severe combined immunodeficient (SCID) mice model.^203^ Combining in vitro confirmation of proteasome-dependent splicetope with screening of specific anti-tumor CD8^+^ T cells enables monitoring of HLA class I binding and immune recognition processes, which will help to obtain more novel tumor-associated splicetope.^200^ Epitopes such as FGF-5, SP110, and gp100-derived splicetope have been identified during in vitro PCPS approach, which could be recognized by CD8^+^ T cells. However, the current research strategy to discover new tumor-specific splicetope needs to be further developed and refined in the future. Protein splicing-derived neoantigens could provide more yet-to-be-developed or identified neoantigens for anti-tumor vaccines and cancer immunotherapy.^38,199^

Most tumor antigens require proteasome processing and TAP-mediated peptide transport. However, most tumors eventually acquire drug resistance and immune escape. It has been reported that tumors can avoid recognition by T cells by producing defective HLA-I antigen processing pathways or downregulating related gene expression. Notably, a class of neoantigens called T cell epitopes associated with impaired peptide processing (TEIPP) have been identified in some HLA-I low/TAP-deficient tumors. They are a class of unmutated antigens derived from the tumor’s own housekeeping proteins that activate TEIPP-specific CD8^+^ T cells and specifically kill these TAP-deficient cancer cells. It is currently believed that TEIPP peptides are immunogenic because they cannot be presented by normal cells, and TEIPP-specific T cells are not negatively selected in the thymus. A TEIPP peptide derived from Lass5 protein, also known as Trh4, was able to activate specific T lymphocytes and inhibit the growth of MHC-I low/TAP-deficient tumors in a TCR transgenic mouse model. In addition, several TEIPP non-mutated tumor epitopes have been identified in humans, including the procalcitonin (ppCT) signal peptide (ppCT16-25, ppCT9-17) regions, and the procalcitonin (pCT) precursor protein (ppCT50-59 and ppCT91-100) regions. Further studies confirmed that these TEIPP-based antigenic peptides can effectively induce anti-tumor CTL effects and inhibit tumor growth. Therefore, targeting these TEIPP neoantigens will potentially provide a promising new immunotherapeutic approach for the treatment of TAP-deficient/HLA-I-low tumors.^27,183,184,204–207^

### Viral-derived tumor antigens (Viral ORFs)

Viral proteins may be considered as another class of neoantigens in tumors caused by viruses because they are almost completely different from normal cellular proteins, and they can elicit high-affinity TCR responses. Some solid tumors are directly caused by viral infection, including Merkel cell carcinoma (MCC) caused by Merkel cell polyoma virus (MCPyV) infection and nasopharyngeal carcinoma caused by Epstein-Barr virus (EBV) infection.^208–213^ In other tumors, viral genes with oncogenic properties can integrate into the cellular genome, promoting the continuous expression of viral genes and leading to tumorigenesis. For example, the expression of E6 and E7 genes from HPV promotes the development and progression of human papillomavirus (HPV)-related cervical, anal, head and neck cancers.^214–217^

Numerous immunotherapy studies have focused on virus-derived tumor antigens. Two of nine HPV-positive patients with metastatic malignancies achieved sustained tumor regressions in ACT research using TILs chosen for their reactivity against viral antigens.^218^ A further investigation revealed that the number of HPV-reactive cells in the reinfused product exceeded those that recognized other types of tumor antigens.^219^ In two separate clinical trials, autologous T cells transduced with anti-E7 TCR responded in 4 of 12 patients, while T cells transduced with anti-E6 TCR responded in all 12 patients.^220,221^ The NCT02280811 and NCT02858310 trials using these TCRs are currently ongoing and should yield more conclusive proof about the value of focusing on HPV epitopes. Treatment of the corresponding tumors with ACT therapy targeting MCPyV and EBV also achieved clinical results, although other effective therapies were also administered in these experimental regimens. Notably, none of these clinical trials occurred with any apparent toxicity to normal tissues. Collectively, these trials demonstrate the safety and efficacy of targeting oncogenic viral proteins to treat related tumors, supporting the development of further comprehensive treatment regimens. Given their critical function in oncogenesis and the fact that patients share them, these neoantigens continue to be desirable targets for cancer immunotherapy.^21,221–223^

The neoantigens are generated as a result of alterations at genomic, transcriptomic and proteomic levels (Fig. 2). Current studies mainly focus on SNVs and INDELs, the most prevalent types of mutations at the genome level in tumor cells. However, the clinical application of neoantigens produced from SNVs and INDELs is limited by their patient specificity and poor immunogenicity, which results in less clinical benefit for cancer patients. Accumulating evidence suggests that alternative sources of cancer neoantigens, such as gene fusions, alternative splicing variants and PTMs, may be attractive novel targets for immunotherapy. The neoantigens produced by gene fusion, particularly the frameshift fusion, have better immunogenicity than the SNV- and INDEL-neoantigens, which were included in numerous clinical trials. Furthermore, neoantigens generated from gene fusion, recurrent mutations in cancer driver genes, non-coding regions and abnormal PTMs have a higher likelihood of being shared among patients, providing readily public neoantigens for immunotherapy.^27,35,38^

## Identification, prediction, and validation of immunogenic neoantigens

Identification of immunogenic neoantigens from the numerous sources mentioned above is a crucial step in the development of effective immunotherapies.^177^ Neoantigens may now be thoroughly screened across the entire cancer spectrum thanks to the convergence of whole-exome sequencing (WES), RNA-seq, and proteomic data from TCGA.^120,224^ However, given the wide variations in tumor types, tumor lesions, and patients, customized immune treatments necessitate the detection and prediction of neoantigens based on distinct patient and tumor characteristics. The identification of genome-expressed mutations as well as details on MHC types of patients are required for the prediction of immunogenic neoantigens, as the sequential stimulation of immune response by tumor neoantigens from mutations depends on several variables, including the translation and processing of peptides, the presentation of the mutated peptides by the MHC molecules and the affinity of the pMHC complexes with the TCRs.^177,225,226^ Two main strategies for identifying neoantigen epitopes are developed: the immunogenomic approach can create virtual peptidomes by in silico methods based on NGS, and the immunopeptidomic strategy use MS to analyze the MHC-loaded peptides.^227^ Several TCR-guided neoantigen discovery strategies have recently been developed to systematically map the immunogenic neoantigens.

### Identification of somatic mutations

The immunogenomic strategies were greatly hastened by comparing the genetic changes between tumor and normal tissue using NGS. Currently, the initial stage in the process of detecting possible neoantigens from NGS data is mapping tumor-specific genetic abnormalities using WES of the tumor and normal DNA. RNA-seq data may be combined with WES to determine whether a mutant gene is expressed in the tumor. In addition, more hidden biological information, such as information about copy number changes, microbial contamination, transposable elements, cell type, and the existence of neoantigens, can be found in RNA-seq.^228,229^ RNA-seq can also be used to detect alternative splicing events and estimate the relative frequency of the mutant allele’s expression.^230^ By using methods like mate-pair sequencing that may detect chromosomal rearrangements, the predictive values of NGS-based TMB measures may be greatly improved.^110^ Recent studies have shown that antigenic peptides are produced by transcripts with frameshift mutations and atypical splicing patterns when NMD is assumed to be present. Exact peptide sequences from full-length transcript structures are required in order to fully identify the neoantigens that resulted from frameshift mutations and aberrant isoforms.^231^ Using the Oxford Nanopore Technologies nanopore-type sequencer MinION, full-length transcriptome sequencing may cover the whole transcript at the proper sequencing depth with an accuracy of roughly 90%, providing complementary information to the current RNA-seq to identify allele-specific transcription and splicing.^143,232^

Based on cancer genomic data, the immunogenomic technique predicted millions of possible mutation-derived neoantigens, but the vast majority of them did not manifest in proteomic profiling of HLA-bound peptides.^233,234^ The high-throughput identification of peptides attached to MHC is made possible by immunopeptidomics techniques, which use MS to directly examine the immunoprecipitated and extracted MHC-bound peptides.^230,235–239^ MS has advanced in verifying in silico predicted neoantigens.^38^ Comparing the tandem mass spectra of the sample with that of the synthetic peptide can verify the neoantigens that are predicted by immunogenomic approaches.^240,241^ Particularly for rare HLA allotypes and HLA-II ligands, mapping the tumor HLA ligandome has helped to uncover targets for the neoantigen-specific cancer immunotherapies in clinical trials.^38^ In addition to validate the neoantigens arising from aberrant DNA sequence or RNA expression, MS-based proteomics provide the "gold standard" for neoantigen detection at the protein level, which cannot be discovered from DNA and RNA studies. For instance, MS can be used to detect novel MHC-associated neoantigens resulting from PTMs that are dysregulated during cellular transformation.^127,188,190,242–244^ Moreover, MS is also integrated with NGS to further detect the tumor-specific neoantigens created by somatic mutations, non-coding RNA and proteasome splicing, which is omitted by whole-exome or transcriptome-based sequencing technology.^38,172,236^ To allow a deeper knowledge of neoantigens in protein levels, more user-friendly and practical tools that integrates genomic, transcriptomic and proteomic data for immunopeptidomic-based neoantigen detection should be created.

### In silico neoantigen prediction

**Fig. 3:**
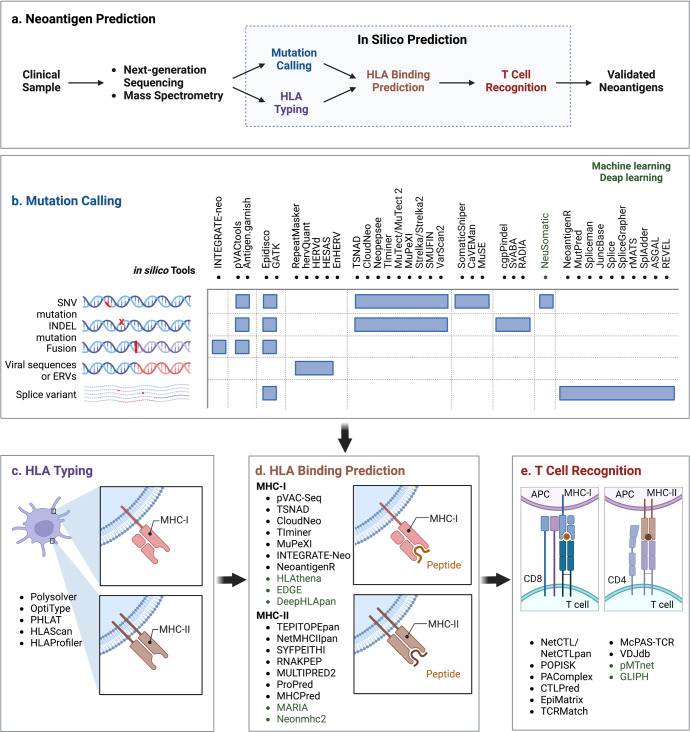
Computational workflow for neoantigen prediction. Current available bioinformatic pipelines for neoantigen prediction from somatic mutations share four main computational modules: (i) HLA typing from tumor WGS, WES data and RNA-seq; (ii) mutant peptide calling using a set of somatic mutations and splicing variants; (iii) HLA binding prediction; and (iv) T cell recognition prediction. The in silico tools for mutation calling are listed as follows. Mutation calling: INTEGRATE-neo,^561^ neoFusion,^80^ pVACtools,^562^ Epidisco,^563^ GATK^564^ and Antigen.garnish,^565,566^ Spliceman,^567^ MutPred,^568^ REVEL,^569^ rMATS,^570^ pVACseq,^240^ Neopepsee,^571^ MuPeXI,^572^ RepeatMasker, CloudNeo,^573^ Tlminer, MuTect/MuTect2, Strelka/Strelka2,^574^ SMUFIN, VarScan2, SomaticSniper, CaVEMan, MuSE, cgpPindel, SvABA, RADIA, NeuSomatic, NeoantigenR, MutPred, JuncBase, Splice, SpliceGrapher, rMATS, SplAdder, ASGAL, REVEL, TSNAD,^575^ HERVd,^569^ HESAS^576^ and EnHERV,^577^ hervQuant.^578^ HLA typing: Polysolver,^254^ OptiType,^253^ HLAreporter,^579^ PHLAT,^580^ HLAScan,^260,581^ HLAProfiler.^260^ HLA binding affinity: NetMHCpan,^265^ NetMHCIIpan4.0,^267^ MixMHC2pred,^582^ MARIA,^268^ neomhc2,^583^ pVAC-Seq, TIminer, HLAthena, DeepHLApan, TEPITOPEpan, NetMHCIIpan, SYFPEITHI, RNAKPEP, MULTIPRED2, ProPred, MHCPred, MARIA, Neonmhc2, EDGE.^38,238^ T cell recognition: NetCTL/NetCTLpan, POPISK, PAComplex, CTLPred, EpiMatrix, TCRMatch

Based on the NGS data, virtual peptidomes have been created and potential neoantigens have been discovered by in silico methods.^177,245^ Briefly, a typical workflow for neoantigen prediction can be summarized into the following steps: (i) mutation calling, (ii) HLA typing, (iii) neoantigen filtering and prioritization based on HLA binding affinity, and (iv) experimental validation of immunogenic neoantigens using T cell-based assays (Fig. 3).^177,246,247^

### HLA typing

Neoantigens are often presented in a cell-specific way by MHC-I for CD8^+^ T cells and MHC-II for CD4^+^ T cells, much like other antigens. Humans have more than 24,000 distinct HLA-I (HLA-A, -B, and -C) and HLA-II (HLA-DR, HLA-DQ, and HLA-DP) alleles, and their admixture results in polymorphism diversity.^248–251^ The HLA alleles of the patient determine their tumor-specific neoantigen repertoire that will be presented for T cell recognition. In addition, HLA-LOH, which occurs in 40% of NSCLC, impairs the presentation of neoantigens, facilitating immune evasion. Therefore, one of the most important initial steps in neoantigen prediction is determining the patient’s HLA genotypes.^83,252^ Several computational methods can now be applied with NGS data to achieve this goal. Most methods rely on DNA-derived NGS data acquired from WES or WGS. For example, Optitype^253^ and Polysolver^254^ are well performing tools for identification of class I HLA alleles. A bioinformatics tool, LOHHLA, is developed for accurate measurement of allele-specific HLA copy numbers. The tools, including HISAT genotype,^255^ ATHLATES,^256^ and HLA-HD^257^ can be used for both class I and class II typing.^83^ RNA-seq data can also be used by tools, such as arcasHLA,^258^ seq2HLA^259^ and HLAProfiler,^260^ to type HLA alleles with advantage of the unbiased dataset that covers both fully expressed parental alleles equally.^260^ The newly developed RNA-seq data-based methods bring a new dimension to HLA typing and biomarker investigations, even though Optitype discovered that WES produced superior results for HLA typing than RNA-seq data.^230,253^

### Mutation and variant calling

By comparing NGS data of tumor and normal tissues from the same patient, mutant peptides resulting from somatic mutations can be predicted.^261^ WES is the recommended source of NGS data for neoantigen prediction because it offers the highest mutation coverage through focusing on the protein-coding regions of the genome. The computational analysis consists of data pre-processing and quality control, variant calling for somatic mutations, and prediction of the altered proteins and functional impact utilizing public genomic, transcriptomic, and proteomic sequence databases. For various neoantigen sources, a variety of integrated techniques have been developed for neoantigen identification and prioritization.^29^ Based on the strategy employed to screen putative neoantigens, these technologies can be classified into two groups: stepwise-analyses-based filtering strategy and integrative-scoring-system-based filtering strategy. The efficient one-stop tools accept WES/WGS and RNA-seq data as input and perform a series of filtering steps based on selected cutoff metrics, such as the binding affinity of peptides and MHC molecules, sequence coverage, variant allele frequency and gene expression, to remove false positives and generate a list of potential neoantigens. An integrated scoring system-based filtering technique assesses the immunogenicity of neoantigens by a quantitative score based on significant neopeptide characteristics, including the rank affinity of the mutant and normal peptides, the frequency of mutant alleles, and the amount of gene expression to experimentally assess the immunogenicity of the discovered neopeptides.^38,262,263^ Recently, a scoring method for evaluating immunogenicity that is based on machine learning models has also been suggested, optimizing the accurate prediction of neoantigens and reducing false positives.^177^ For a review and extensive discussion of these methods, we refer to prior literatures.^246,249^

### Prediction of HLA binding and neoantigen presentation

Numerous computer prediction tools have been created for the in silico discovery of neoantigens based on MHC molecule processing and presentation, including NetChop, NetCTL and NetCTLpan^264,265^ (Fig. 3). The prediction capacity is actively improved by incorporating HLA-ligandome data into machine learning algorithms, such as linear regression and artificial neural networks.^230,249^ In vitro peptide-HLA binding dataset is used to train machine learning models by NetMHCpan^265^ and MHCflurry^266^ that are the main component of current HLA ligand identification pipelines.^38^ It is noteworthy that NetMHCpan, in contrast to state-of-the-art methods, improves the prediction performance of tumor neoantigens by combining information from binding affinity data with MS peptidome data to give a "panspecific" machine-learning strategy for MHC-I alleles.^230,264,267^ Two recent studies created computational frameworks called MSIntrinsic and EDGE that are highly effective in predicting HLA antigens using HLA peptides acquired from RNA-seq and liquid chromatography tandem MS (LC-MS/MS) data. Based on 24,000 HLA-I peptides collected by LC-MS/MS, the neural-network prediction algorithm, MSIntrinsic, outperformed previous affinity-based predictors by an average of 30% in positive predictive value (PPV).^251^ Similar findings were made by EDGE, which found that adopting a deep-learning architecture to identify HLA ligands using proteomic and transcriptomic data can improve the accuracy of HLA antigen prediction by up to ninefold.^38,238^

Emerging evidence has proved the significance of MHC-II neoantigens in anti-tumor immune response.^234,268–271^ A wide range of computational techniques for predicting MHC-II binding epitopes have been developed using artificial neural networks, including NetMHCII, NetMHCIIpan,^272,273^ SYFPEITHI, RNAKPEP, MULTIPRED2, ProPred, and MHCPred. However, compared to MHC-I molecules, computational prediction of the MHC-II-peptide binding affinity are currently less precise. First, compared to MHC-I molecules, MHC-II-binding peptides are more promiscuous in terms of peptide length and binding sequence motifs. Second, the polymorphism of the α and β chains in MHC-II molecules also considerably expands the diversity of peptide binding specificity.^38,230^ Recently, computational methods based on transcriptome and MS data have been developed. The deep learning model trained by MARIA, which incorporates both sequencing data with naturally occurring MHC-II ligandomes, was demonstrated to outperform the most widely used predictor NetMHCIIpan3.1 in the lymphoma dataset when cross validated against known MHC-II ligands. However, more study using significant datasets is necessary to demonstrate its robustness and effectiveness.^38,268^

Given multiple processes control the neoantigen presentation, it can be inferred that improving binding affinity alone does not accurately reflect cellular processing and CD8^+^ T cell responses. Additional properties, including proteasomal cleavage, transportation of peptides into the endoplasmic reticulum, and HLA alleles, are in conjunction with binding affinities between the peptide and the MHC molecules to prioritize possible neoantigens.^230^

### Evaluation and validation of candidate neoantigens’ immunogenicity

It is well known that an immunogenic neoantigen must satisfy two or more requirements, the main bottlenecks are appropriate MHC molecule presentation and effective TCR recognition. According to recent studies, the majority of predicted neoantigens via MHC molecule presentation do not trigger an immune response.^234,274,275^ Therefore, while assessing the immunogenicity of potential neoantigens, it is crucial to take the TCR recognition of pMHC complexes into account. There are many in silico techniques that can forecast neoantigen-specific T cell recognition. The most used method is NetCTL/NetCTLpan, which generates a composite score rather than predicting T cell binding directly by combining MHC binding, C-terminal cleavage affinity and TAP transport.^38^ Recent studies use machine learning or deep learning techniques to predict TCR-peptide/-pMHC binding. The batch of TCR repertoire annotation in several manually curated databases, including McPAS-TCR and VDJdb, allows for the training of TCR specificity predictors and match against TCRs of interest.^276–278^ McPAS-TCR provides a list of TCR sequences linked with various pathologies, while VDJdb offers a detailed description of TCR:pMHC interactions based on epitope-centric approach for TCR annotation rather than the underlying biological context.^279,280^ Besides identification of TCR-pMHC pairings, clustering methods, like pMTnet and GLIPH, can also cluster TCRs that recognize the same epitope and predict their HLA restriction.^281–285^ Nevertheless, the prediction for binding affinity of TCR and pMHC in silico is still challenging due to the low affinities of TCRs for their pMHC ligands.^230,246,249,286,287^

For a more precise assessment of the possible application of neoantigens in immunotherapy, experimental validation of their T cell reactivity is essential. Neoantigen-reactive T cells have been validated or screened using T cell-based assays, multicolor-labeled MHC tetramers, the enzyme-linked immunosorbent spot (ELISpot) and T-cell repertoire profiling.^33,288^ T cell immunogenicity assay is the most direct way to evaluate the immunogenicity of candidate neoantigens. The entire set of possible mutant peptides discovered by cancer exome/RNA-seq can be tested using T cells from either cancer patients or healthy donors. After peptide stimulation, the in vitro expanded neoantigen-specific T cell reactivity is measured by flow cytometric measurement of the T-cell activation markers 4-1BB and OX-40 and IFN-production on the ELISpot assay.^62,289^ Multicolor-labeled MHC tetramers allow for the highly sensitive and minimally material-required evaluation of T cell reactivity against a wide range of potential epitopes using DNA barcoding, lanthanide coding, or fluorochrome coding of peptides. These technologies rely on epitope predictions and are low throughput since they can only efficiently generate a subset of the human MHC class I alleles. Integrating single-cell RNA sequencing (scRNA-seq) with TCR sequencing of responsive cell groups may be used to boost the sensitivity of detection. The scRNA-seq was used to discover paired TCR sequences linked with cells expressing high levels of IFN- γ and IL-2 in TILs co-cultured with tandem minigene (TMG)-transfected or peptide-stimulated antigen-presenting cells (APCs).^290,291^ Based on WES-guided prediction of neoantigens and TCR sequencing of short-term peptide-stimulated T cell cultures, the Mutation-Associated Neoantigen Functional Expansion of Specific T cells (MANAFEST) assay sensitively characterizes neoantigen-specific TCR Vβ clonotypes. The MANAFEST assay is compatible with all HLA haplotypes and can track neoantigen-specific T cells in formalin-fixed paraffin-embedded (FFPE) and/or frozen tissues. In addition to assess the tumor specificity of TCR Vβ clonotypes, MANAFEST can also look into the dynamics of the neoantigen-specific T cell response over time and monitor the efficacy of immunotherapy using liquid biopsies obtained before or after treatment.^292^

Several unbiased TCR-guided neoantigen discovery strategies have been developed to systematically profile neoantigen-specific TCRs. A yeast-displayed pMHC library can be used to discover neoantigen-specific TCRs. However, it is a time-consuming process to make soluble TCR reagents. Without endogenous processing of neoantigens or functional activation of T cells, the identified random peptides may not represent the physiological TCR-pMHC interaction.^293^ To overcome these drawbacks, two innovative strategies make use of different biological processes to mark the target cells in a co-culture system. One approach utilizes the chimeric receptors known as signaling and antigen-presenting bifunctional receptors (SABRs), which can induce a TCR‐like signal following pMHC-TCR interactions. SABRs enable the successful identification of TCR-pMHC interaction, which can be used for both known public TCRs and private neoantigen-specific TCRs.^294^ Trogocytosis, a membrane transfer process, is exploited by a cell-based selection platform for TCR ligand discovery. The TCR-pMHC interactions result in specific labeling of cognate target cells, which are then isolated and sequenced to identify the neoantigen-specific TCRs.^295^ In addition, putative pMHCs are displayed on spectrally encoded beads in BATTLES, facilitating the investigation of neoantigen-specific T cell responses under physiological force.^296^ T-Scan, a method for TCR epitope scanning independent of predictive algorithms, relies on the physiological activity of T cell killing rather than just assessing TCR-pMHC binding affinity, enabling the interrogation of a significantly larger antigen space than previous methods.^297^ Thus, these emerging approaches for TCR ligand discovery will be useful for studying the immunogenicity of candidate neoantigens, providing new targets for immunotherapy.

## Neoantigens-based therapeutic strategies

**Fig. 4:**
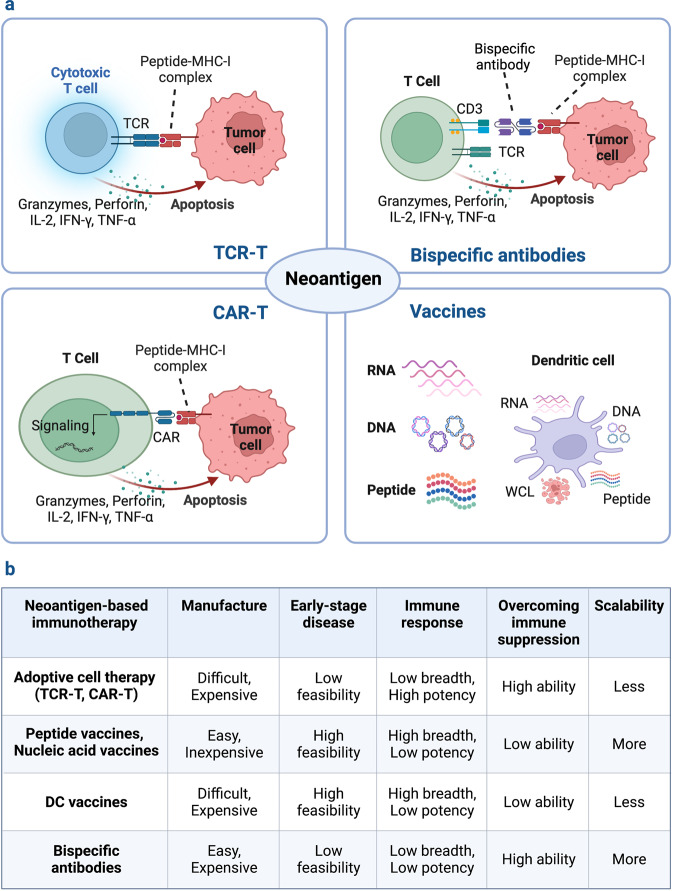
Classification of neoantigen-based therapies. Immunotherapies that target neoantigens mainly include ACTs, bispecific antibodies and cancer vaccines. Cancer vaccines stimulate a specific immune response to tumor neoantigens using nucleic acids, peptides and DCs. The ACT utilizes the neoantigen-specific TCR or CAR engineered T cells to selectively recognize and kill tumor cells. The bispecific antibodies have one arm that targets neoantigens presented by tumor cells and one arm that targets CD3 on the surface of T cells

As previously mentioned, tumor-specific neoantigens arising from genetic alterations elicit high-avidity T cells due to the absence of thymic selection and central tolerance. Based on their advantages of tumor-specific and immunogenetic, neoantigens may serve as emerging targets for cancer immunotherapies, including tumor vaccines, ACTs and antibody-based therapies, as well as potential predictors for ICBs (Fig. 4).^8,226,298,299^ The neoantigens consist of either personalized neoantigens found specifically for each patient or shared neoantigens expressed in numerous patient cancers. The off-the-shelf therapies based on public neoantigens are less resource- and time-intensive than individualized neoantigen therapies. Because personalized neoantigens are patient-specific, they cannot be used to target a large number of patients. With the recent advance in high-throughput sequencing, personalized neoantigens enable the immune system to target appropriately immunogenic epitopes on malignancies without predefined public antigens.^300,301^

### Neoantigen-based therapeutic vaccines

**Fig. 5:**
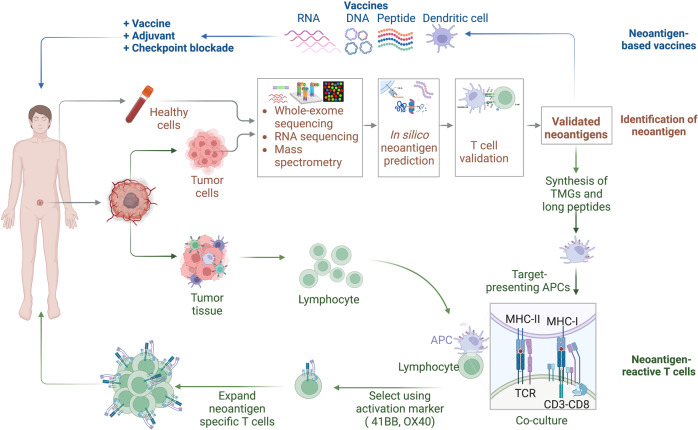
Schematic illustration of neoantigen-based cancer immunotherapy production. The individualized neoantigens are identified using blood cells and tumor tissues from patient. These patient-specific neoantigens are used to develop immunotherapies, such as cancer vaccines and ACTs. Cancer vaccines in the form of peptides, DNA or mRNA, and dendritic cells are generated and administered to the same patient. For ACTs, T cells are extracted from the peripheral blood or tumor tissues of a patient and then induced to proliferate by cytokines, monoclonal antibodies against CD3 and CD28, and other reagents. The development of neoantigen-specific T lymphocytes with neoantigen-specific targeting requires co-culturing T cells with primed APCs and genetic engineering of immune cells with TCRs or CARs. After sufficient T cell expansion, T cell products are injected into lymphodepleted patients with the hope of eliciting an immune response that attacks the tumors

Neoantigen vaccines are an effective approach for stimulating, enhancing, and diversifying anti-tumor T cell responses, with their high feasibility, general safety and easier to manufacture. Various forms of neoantigen-based vaccines, such as peptide, nucleic acid and dendritic cell (DC) vaccines are being evaluated in clinical trials on patients with different types of tumors (Fig. 5).^9,15,245,302^ Current peptide and nucleic acid vaccines mainly target the predicted neoantigens derived from somatic mutations, including SNVs, frameshift INDELs and gene fusions. DC vaccines can target both selected neoantigens via pulsing with synthetic peptides or nucleic acids and overall TSAs by introducing with whole cell lysates (WCL).

### Peptide vaccines

Peptide-based neoantigen vaccines have received most of the attention in the research area of personalized neoantigen vaccines due to their high specificity, economical manufacture and established safety record (Table 3).^177,303,304^ The neoantigen peptides can be produced as genetically encoded long peptides or fused polypeptides and chemically synthesized short peptides. The peptides are subjected to affinity chromatography, size-exclusion chromatography (SEC) or high-pressure liquid chromatography (HPLC) to obtain sterile, endotoxin-free products with a purity of >98%. Following verification by MS, the peptides are mixed with appropriate adjuvants for subcutaneous injection immunization.^99,305^ In a phase I immunotherapy clinical trial in patients with disseminated synovial sarcoma, an SYT-SSX neoantigen peptide-based vaccine prevented disease progression in one patient and successfully induced specific CTL responses in four patients, and no serious adverse reactions or delayed-type hypersensitivity (DTH) reactions occurred throughout the treatment.^305^ The peptides, such as KQSSKALQR, produced from the breakpoint of BCR-ABL can be processed in the cytosol and loaded onto MHC molecules, which will be transferred to the CML cell surface for potential T cell recognition.^86^ In an initial clinical trial, this neoantigen-based vaccine elicits a BCR-ABL peptide-specific T cell immune response, while has no significant toxic effects.^99,306^ Selected neoantigens containing T cell epitopes can be produced in the form of single epitopes, polypeptide chains, or peptide pools. To overcome issues like tumor heterogeneity, HLA haplotype diversity and antigen down-regulation, overlapping peptides or long multi-epitope peptides rather than short single-epitope peptides are typically used to stimulate a powerful immune response in T cells.^307^ In addition, immunostimulatory adjuvants and multimeric formulation techniques are being developed to boost the immunogenicity of personalized peptide vaccinations. Therefore, personalized neoantigen vaccines based on synthetic peptides have been evaluated in clinical studies on patients with various types of cancers, including lung cancer, breast cancer, bladder cancer, pancreatic cancer, pediatric brain tumor, melanoma, and colorectal cancer (Table 4).

**Table 3:** Advantages and disadvantages for neoantigen-based immunotherapies

Immunotherapy	Formulation	Advantages	Disadvantages
Adoptive cell therapy	Tumor-infiltrating lymphocytes (TILs)	High specificity, low toxicity; direct and continuous killing of target cells	Expensive, time- and labor-intensive process; MHC-restricted; high proportion of tumor unrelated bystander TILs
	T cell receptors engineered T cells (TCR-T)	Recognize intracellular neoantigens with a high mutation rate; poor affinity but high neoantigen sensitivity; natural protein with low immunogenicity	Expensive, time- and labor-intensive process; MHC-dependent neoantigen presentation; non-specific efficacy due to mispairing with endogenous TCRs; limited patient applicability, especially in low mutation rate cancers
	Chimeric antigen receptor T cells (CAR-T)	MHC-independent neoantigen detection; high neoantigen affinity; precisely controlling of neoantigen response by modular design; recognizing proteins, carbohydrates and glycolipids	Expensive, time- and labor-intensive process, toxic effects; unnatural protein with potential immunogenicity; limited recognition of cell-surface neoantigen due to competing soluble neoantigen; on-target CAR-T cell activation by soluble antigens
Vaccine	Peptide vaccines	Inexpensive and easy to produce; high specificity; low toxicity	Restricted to the HLA subtype; low/moderate immunogenicity
	Nucleic acid vaccines (RNA vaccine and DNA vaccine)	Inexpensive to produce; easy delivery of multiple antigens; not restricted to HLA-patient type; activation of both cellular and humoral immunity	Poorly immunogenic in humans; RNA vaccines require specific transportation or storage conditions
	Dendritic cell (DC) Vaccines	High immunogenicity; control of antigen presentation	Expensive and difficult to produce; risk of leukapheresis (vascular injury, electrolyte imbalance)
Antibody-based therapy	Full-length antibodies, antibody-drug conjugates, bispecific antibodies	Synergy helps circumvent drug resistance	Costly for widespread use targeting individual tumor neoantigens

**Table 4:** The clinical studies of neoantigen-based immunotherapies

Neoantigen-based immunotherapies	Therapeutic strategies	Cancer types	Clinical Trail Numbers (Primary Outcome Measures)
Cancer vaccines			
Dendritic cell vaccine	Dendritic cell vaccine	Breast cancer, colorectal cancer, diffuse intrinsic pontine glioma, glioblastoma, gastric cancer, hepatocellular carcinoma, non-small cell lung cancer, esophageal squamous cell carcinoma, esophagus cancer, HPV-positive cancer, melanoma, gastrointestinal cancer, ovarian cancer, pancreatic cancer	NCT04105582 (One-year safety and adverse events (AEs)), NCT04879888 (AEs), NCT01885702 (5-years safety), NCT03914768 (2-years safety and overall survival (OS) at 12 months (OS12)), NCT05317325 (AEs), NCT05023928 (AEs), NCT04147078 (5-years disease-free survival (DFS)), NCT03674073 (One-year safety and AEs), NCT03870113 (AEs and immunogenicity), NCT03300843 (Clinical response rates), NCT03871205 (AEs and immunogenicity), NCT02956551 (AEs), NCT04078269 (Safety and tolerability), NCT03205930 (AEs), NCT05270720 (AEs), NCT01278940 (Safety and toxicity)
	Dendritic cell vaccine + ACTs	Melanoma, bladder cancer, colorectal cancer	NCT05235607 (AEs)
	Dendritic cell vaccine + chemical drug	Advanced biliary tract malignant tumor, acute myeloid leukemia, acute lymphocytic leukemia, chronic myelogenous leukemia, myelodysplastic syndrome, non-hodgkin’s lymphoma, glioblastoma multiforme	NCT02632019 (2-years OS), NCT00923910 (AEs), NCT04968366 (AEs)
	Dendritic cell vaccine + ICBs	Hepatocellular carcinoma, colorectal cancer liver metastases	NCT04912765 (2-years relapse free survival)
	Dendritic cell vaccine + chemical drug + ICBs	Lymphocytic leukemia	NCT03219450 (One-year safety)
	Dendritic cell vaccine + TILs	Fallopian tube cancer, ovarian cancer, primary peritoneal cancer	NCT03735589 (AEs and dose-limiting toxicities (DLTs))
DNA vaccine	DNA vaccine	Glioblastoma, melanoma, pancreatic cancer, pediatric recurrent brain tumor, lynch syndrome, non-small cell lung carcinoma	NCT04015700 (Safety and tolerability), NCT03655756 (Serious adverse events (SAEs) and DLTs), NCT03122106 (Safety), NCT03988283 (Safety and tolerability), NCT05078866 (AEs and immunogenicity), NCT04990479 (Safety and tolerability)
	DNA vaccine + ICBs	HPV 16-positive oropharynx cancer, small cell lung cancer, prostate cancer, renal cell carcinoma, triple-negative breast cancer, solid tumors, hepatocellular carcinoma	NCT04001413 (5 years clearance rates of HPV), NCT04397003 (Safety and tolerability), NCT03532217 (Safety, tolerability, and clinical response rates), NCT03598816 (Safety), NCT03199040 (Safety), NCT04251117 (AEs and immunogenicity), NCT05354323 (AEs)
mRNA vaccine	mRNA vaccine	Melanoma, colon cancer, gastrointestinal cancer, genitourinary cancer, hepatocellular cancer, esophageal cancer, non-small cell lung cancer, triple negative breast cancer	NCT02410733 (AEs), NCT03480152 (Clinical response rates and AEs), NCT05198752 (DLTs), NCT02035956 (Safety and tolerability), NCT03908671 (AEs), NCT02316457 (AEs)
	mRNA vaccine + ICBs	Melanoma, non-small cell lung cancer, bladder cancer, colorectal cancer, triple negative breast cancer, renal cancer, head and neck cancer, pancreatic cancer, solid tumors	NCT03289962 (DLTs and AEs), NCT04267237 (62-months DFS), NCT03897881 (3-years recurrence-free survival (RFS)), NCT03948763 (DLTs and AEs), NCT03313778 (AEs)
	mRNA vaccine + ICBs + Chemical drug	Pancreatic cancer	NCT04161755 (Toxicity)
Peptide vaccine	Peptide vaccine	Melanoma, non-small cell lung cancer, pancreatic cancer, pediatric brain tumor, colorectal cancer, breast cancer, head and neck squamous cell carcinoma, liver cancer, diffuse intrinsic pontine glioma, glioblastoma, glioblastoma multiforme, astrocytoma, acute lymphoblastic leukemia, esophageal cancer, lynch syndrome, bladder urothelial carcinoma	NCT03662815 (Clinical response rates and AEs), NCT01970358 (AEs), NCT04509167 (WBC and RBC changes), NCT04397926 (Safety), NCT05013216 (Toxicity), NCT03956056 (Safety), NCT05111353 (Safety), NCT03068832 (Safety and tolerability), NCT04087252 (Safety), NCT05238558 (Delayed type hypersensitivity (DTH), proliferative T-cell responses and AEs), NCT03552718 (AEs), NCT04749641(Safety and one-year survival rate), NCT05356312 (NA), NCT02510950 (Safety and tolerability), NCT03559413 (Clinical response), NCT02992977 (AEs), NCT05307835 (One-year relapse-free survival and AEs), NCT04943718 (AEs), NCT03807102 (AEs and 2-years DFS), NCT03715985 (AEs), NCT04998474 (Immunogenicity), NCT04810910 (AEs and 4 years-relapse free survival), NCT03673020 (AEs), NCT03645148 (Objective response rate (ORR) and AEs), NCT03558945 (2-years overall survival)
	Peptide vaccine + ICBs	Breast cancer, colorectal cancer, pancreatic cancer, melanoma, advanced solid tumor, diffuse intrinsic pontine glioma, diffuse midline glioma, non-small cell lung cancer, head and neck squamous cell, fibrolamellar hepatocellular carcinoma, follicular lymphoma, glioblastoma, hepatocellular carcinoma, urothelial carcinoma, renal cell carcinoma, bladder urothelial cancer, gastrointestinal tract cancer, myeloproliferative neoplasms, squamous cell lung cancer, gastroesophageal adenocarcinoma, urothelial carcinoma, ovarian cancer, platinum-resistant fallopian tube carcinoma, primary peritoneal carcinoma	NCT04864379 (ORR and AEs), NCT05269381 (AEs), NCT05098210 (AEs), NCT03606967 (6- and 12-months progression-free survival (PFS)), NCT02600949 (AEs), NCT03597282 (AEs), NCT04072900 (AEs and Clinical response), NCT04117087 (AEs), NCT04943848 (Safety and Tolerability), NCT03633110 (AEs), NCT04248569 (Toxicity), NCT03361852 (Clinical response), NCT03422094 (Safety and Tolerability), NCT03568058 (AEs), NCT03359239 (AEs), NCT05153304 (AEs), NCT02950766 (DLTs), NCT03929029 (DLTs), NCT04930783 (DLTs), NCT04364230 (Safety and immunogenicity), NCT05444530 (DLTs and AEs), NCT03166254 (Safety), NCT04266730 (AEs), NCT03639714 (AEs, SAEs, DLTs and ORR), NCT03953235 (AEs, SAEs, DLTs and ORR), NCT04024878 (AEs and SAEs), NCT04799431 (Toxicity), NCT03206047 (AEs and one-year PFS), NCT02897765 (AEs and SAEs)
	Peptide vaccine + chemical drug	Non-small cell lung cancer, smoldering plasma cell myeloma	NCT04487093 (AEs), NCT03631043 (AEs)
	Peptide vaccine + ICBs + chemical drug	Non-small cell lung cancer	NCT03380871 (AEs and SAEs)
	Peptide vaccine + ICBs + chemical drug + Radiotherapy	Glioblastoma	NCT02287428 (AEs)
Adoptive cell therapy (ACT)			
TCR-T cell	TCR-T cell	Gynecologic cancer, colorectal cancer, pancreatic cancer, non-small cell lung cancer, cholangiocarcinoma, endometrial cancer, ovarian carcinoma, squamous cell lung cancer, adenocarcinoma of lung, adenosquamous cell lung cancer, hepatocellular carcinoma, advanced malignant solid tumor, melanoma, advanced solid tumor	NCT05194735 (DLTs, ORR and AEs), NCT05105815 (18-months DFS), NCT04625205 (AEs and SAEs), NCT03171220 (AEs), NCT05020119 (AEs and DLTs)
	TCR-T cell + chemical drug	Endocrine/neuroendocrine,non-small cell lung cancer, breast cancer, gastrointestinal/genitourinary cancers, ovarian cancer, melanoma, head and neck squamous cell carcinoma, urothelial carcinoma, renal cell carcinoma, small-cell lung cancer, cutaneous squamous cell carcinoma, anal squamous cell carcinoma, merkel cell carcinoma, vaginal cancer, cervical cancer, anal cancer, penile cancer, oropharyngeal cancer	NCT04102436 (Response rate), NCT04596033 (AEs), NCT02280811 (DLTs and ORR), NCT02858310 (Overall response rate and AEs)
	TCR-T cell + ICBs	Malignant epithelial neoplasms, solid tumor	NCT05349890 (AEs and SAEs), NCT04520711 (Safety, tolerability and DLTs), NCT03970382 (DLTs and AEs)
	TCR-T cell + ICB + chemical drug	Endocrine tumors, non-small cell lung cancer, ovarian cancer, breast cancer, gastrointestinal /genitourinary cancers, neuroendocrine tumors, multiple myeloma, Hpv-16 positive squamous cell anal cancer	NCT03412877 (Response rate), NCT04536922 (Treatment effect)
	TCR-T cell + radiotherapy	Hepatocellular carcinoma	NCT03199807 (AEs)
TILs	TILs	Malignant epithelial tumors, malignant solid tumor	NCT05141474 (AEs, SAEs and treatment-limiting toxicity (TLT))
	TILs + chemical drug	Gastric cancer, colorectal cancer, pancreatic cancer, gall bladder cancer, esophageal cancer, recurrence tumor, metastatic cancer, solid tumor	NCT04426669 (Maximum tolerated dose (MTD)), NCT03658785 (ORR), NCT02959905 (AEs)
	TILs + ICBs	Melanoma, advanced non-small cell lung cancer	NCT03997474 (AEs), NCT04032847 (AEs)
	TILs + ICBs + chemical drug	Non-small cell lung cancer, squamous cell carcinoma, adenosquamous carcinoma	NCT03215810 (DLTs)
CAR-T	CAR-T therapy + Chemical drug	Glioblastoma multiforme	NCT02844062 (Safety)
Immune checkpoint blockade (ICB)			
ICBs	ICBs	Acute myeloid leukemia, myelodysplastic syndrome, bladder cancer, melanoma, colon cancer, glioma, glioblastoma, head and neck squamous cell carcinoma, HPV16-positive cancer, locally recurrent cancer, nasopharyngeal carcinoma, non-small cell lung cancer, oesophageal cancer, prostate cancer, rectal cancer, uterine cancer	NCT03600155 (Optimal dose), NCT02553642 (ORR), NCT03827044 (3-years DFS), NCT03718767 (6-months PFS), NCT03925246 (24-weeks PFS), NCT03082534 (ORR), NCT03357757 (Treatment effect), NCT03813394 (2-years ORR and PFS), NCT02437279 (Safety), NCT04825990 (ORR), NCT03130764 (Clinical response), NCT03653052 (Clinical response), NCT02113657 (Clinical response), NCT03040791 (Clinical response), NCT04019964 (Clinical response), NCT04293419 (Clinical response), NCT04262089 (Clinical response)
	ICB + chemical drug	Acute myeloid leukemia, bladder cancer, breast cancer, colorectal cancer, hormone receptor positive tumor, endometrium cancer, gastric cancer, hepatocellular carcinoma, ovarian cancer, triple-negative breast cancer, peritoneal cancer, fallopian tube cancer, prostate cancer, head and neck squamous cell carcinoma	NCT04214249 (Clinical response), NCT03978624 (Clinical response), NCT02990845 (8-months PFS rate), NCT02453620 (AEs), NCT03409198 (3-years toxicity and PFS), NCT05456165 (AEs), NCT05201612 (10-months ORR), NCT03832621 (8-month PFS), NCT03186326 (12-months PFS), NCT04659382 (9-months PFS), NCT04262687 (10-months PFS), NCT05141721 (5-years PFS), NCT04014530 (AEs and ORR), NCT03918499 (Safety), NCT03655002 (DLTs and AEs), NCT03126812 (Clinical response), NCT03554317 (Clinical response), NCT04336943 (Clinical response), NCT04068194 (MTD and ORR), NCT05317000 (Clinical response), NCT02883062 (tumor infiltrating lymphocyte (TIL) percentage)
	ICB + radiotherapy	Cutaneous T cell lymphoma	NCT03385226 (Overall response rate)
	ICB + chemical drug + radiotherapy	Colorectal cancer, meningioma, rectal cancer	NCT03854799 (Rate of complete pathologic response), NCT03604978 (MTD, AEs and ORR), NCT04340401 (Rate of complete pathologic response)

Neoantigen peptide vaccines elicit and amplify anti-tumor immune responses in cancers with either high or low mutational burden. A vaccine formulated with the adjuvant poly-ICLC and a synthetic neoantigen long peptide efficiently activates CD8^+^ T and CD4^+^ lymphocytes in patients with advanced melanoma, NSCLC, or bladder cancer, all of which have high levels of mutations (NCT02897765). This neoantigen vaccine prevents recurrence for 25 months after treatment in four out of six high-risk melanoma patients.^15^ In NSCLC patients who have failed in multiple conventional therapies, personalized neoantigen peptide vaccination triggers specific T cell responses targeting EGFR mutations, including the relatively prevalent mutations L858R and T790M. Accordingly, a large subset of NSCLC patients responding relatively poorly to ICB approaches may benefit from the neoantigen vaccines based on shared immunogenic EGFR mutations.^308^ In addition, the peptide-based neoantigen vaccination can potentially modify the immune milieu of immunologically cold tumors with a relatively low mutational burden, inducing neoantigen-specific T cells to infiltrate and destroy tumor cells. For example, administration of neoantigen vaccines induces T cell immune responses in HLA-A*24:02 or HLA-A*02:01-positive glioblastoma patients. These neoantigen-specific T cells are able to cross the blood-brain barrier (BBB) and infiltrate the tumor, thereby altering the immune milieu of glioblastoma and extending the median overall survival of patients to 29.0 months.^309–314^

Personalized neoantigen peptide vaccines can expand the durability and repertoire of tumor-specific T cells.^30^ According to a retrospective analysis of the circulating immune responses in melanoma patients after vaccination, neoantigen-specific T lymphocytes exhibit a memory phenotype that lasts for an average of ~4 years following vaccination (NCT01970358). The neoantigen-specific T cells have evolved overtime into a variety of clones with different functional avidities. Meanwhile, non-vaccine antigen-directed T cell responses are also detected, suggesting epitope spreading after vaccination. The epitope spreading is associated with prolonged progression-free survival.^15,315,316^ The long-term persistence and diversification of functional neoantigen-specific T cell clones support the neoantigen peptide vaccines as a potent strategy for controlling the continuously evolving metastatic tumors.^317^

The immunogenicity of peptide vaccines can be further enhanced through improving the neoantigen presentation and using immunostimulatory adjuvants.^318–322^ For example, KRAS-G12D mutant peptides are fused to the C-terminal of diphtheria toxin to produce a more immunogenic peptide vaccine. This vaccine boosts CD8^+^ T cells while decreases T regulatory cells in mice with CT26 tumor.^323^ Heat shock proteins (HSPs), like HSP70, have also been complexed with synthetic peptides derived from tumor-specific neoantigens to enhance the presentation and recognition of antigens, which are widely used for treating advanced tumors resistant to conventional therapies (NCT02992977, NCT03673020).^324,325^ Nanoparticle formation is another technique for improving the immunogenicity of peptide vaccines. B16.F10 and CT26 neoantigens formulated with polyethyleneimine (PEI)-adsorbed mesoporous silicas micro-rod (MSR) can completely eradicate existing lung metastases in tumor-bearing mice.^325,326^ Another advantage of nanoparticle platform is capable of co-delivering peptides and adjuvants. Self-assembled intertwining DNA-RNA nanocapsules have been used to efficiently deliver tumor-specific neoantigen peptide and synergistic adjuvants, DNA CpG and shRNA to APCs in lymph nodes. These neoantigen vaccines induce peripheral memory neoantigen-specific CD8^+^ T lymphocyte, suppressing the progression of neoantigen-associated colorectal cancers.^327–329^ High density lipoprotein-mimicking nanodiscs elevate the efficient co-delivery of peptides and adjuvants to lymphoid organs and maintain the neoantigen presentation on DCs. In clinical trials, neoantigen-specific CTLs activated by nanodisc vaccines are 31 times more frequencies than the strongest adjuvant and up to 47 times more than soluble vaccines.^330^ The formulation of SNP-7/8a derived from charge-modified peptide-TLR-7/8a can effectively activate specific CD8^+^ T lymphocytes against 50% of neoantigens with high predicted MHC-I affinity binding, thereby enhancing anti-tumor efficacy.^331^ Collectively, a generic approach can be utilized to improve the anti-tumor immune response of personalized peptide vaccines.

### Nucleic acid vaccines

Like peptide vaccines, nucleic acid vaccines, such as RNA and DNA vaccines, also have the advantage of being low-cost and non-HLA-specific (Table 3). Nucleic acid vaccines can deliver multiple tumor neoantigens in a single vaccination, triggering both cellular and humoral anti-tumor immune responses.^245,262,325^

Currently, mRNA technology has been widely used in the clinical treatment of tumors, the prevention of infectious diseases and protein-encoding therapies. The recent success of the COVID-19 mRNA vaccine has revealed the therapeutic potential of mRNA technology.^332^ mRNA vaccines offer considerable anti-tumor potential due to their advantages in safety, high potency, rapid and low-cost industrial production, and ability to encode entire antigens.^333^ Currently, in vitro transcription (IVT) is the major method used to create mRNA that contains the sequence for neoantigens. A cap structure is added to mRNAs post-IVT to increase their stability and decrease their immunogenicity. After purification through SEC or tangential flow filtration (TFF), appropriate delivery systems, such as liposomes and polymers, are selected to introduce mRNA into cells and tissues to translate the target neoantigens, thereby activating the immune response.^334,335^ Personalized mRNA vaccines based on tumor-specific neoantigens induce a more potent immune response than shared tumor-associated self-antigens due to the absence of central immune tolerance. For example, neoantigen-specific mRNA vaccines in 13 evaluable melanoma patients activated several neoepitope-specific CD4^+^ and CD8^+^ T cells, greatly reducing the cumulative incidence of recurrences and leading to persistent progression-free survival.^302,335,336^ The mRNA-4650 vaccine, which contains defined neoantigens, novel neoantigens derived from driver gene mutations and predicted HLA-I epitopes, elicits both CD8^+^ and CD4^+^ T cell response, with a preference for CD4^+^ T cell responses with no severe side effects.^337^ Clinical studies for the personalized mRNA-4157 and BNT122 vaccines are currently underway. mRNA-4157 monotherapy or in combination with the PD-1 inhibitor is well tolerated and induces a neoantigen-specific T cell response in clinical trials (NCT03313778; NCT03897881).^338^ A phase I trial of RNA vaccine (NCT02316457) in triple negative breast cancer (TNBC) patients demonstrate a highly effective at eliciting robust poly-epitopic T cell responses, increasing the clinical benefit for TNBC patients following surgery and (neo-)adjuvant chemotherapy.^339^ Moreover, the RO7198457 vaccines have been explored by BioNTech to treat various solid tumors, including melanoma, NSCLC and colorectal cancer, in combination with PD-L1 antibody.^340^

mRNA-encoded neoantigen vaccines may offer a proper but more potent immunogenic response and therapeutic efficacy when compared with peptide vaccines. This superiority may arise from the biological function of mRNA as a template for protein synthesis. The mRNA vaccine enables post-translational modification of protein products in human, which has the potential to present various epitopes without being constrained to a specific HLA type. In addition, numerous neoantigen epitopes can be incorporated into the same backbone, producing myriad neoantigens that can exist either as independent molecules or as a series of multiple coding sequences.^302,337^ One such example is the RNA-based poly-neoepitope approach developed by Sahin and colleagues. Ten selected mutations per patient are engineered into two synthetic pharmacologically optimized RNA molecules, each of which encodes five linker-connected 27mer peptides (NCT02035956).^302^ Another example is the personalized cancer vaccines in clinical trials, including mRNA-4157 and mRNA-4650, containing an mRNA backbone that can encode up to 30 different neoantigens.^337^ As a result, mRNA vaccine can express a variety of neoantigens originating from patient’s own tumor, resulting in a stronger immune response.^177^

Effective application of mRNA vaccines in vivo requires maintaining mRNA stability and effective intracellular distribution of the mRNA moiety to target cells. Since RNA is intrinsically unstable, early attempts focused mostly on its stabilization. The 5′ cap structure, the length of 3′ poly(A) tail and regulatory elements in the untranslated regions have all been optimized for this purpose.^177,341^ Efficient intracellular delivery is also required for effective mRNA therapies in vivo. Nanoformulations, such as lipid, calcium, and phosphate nanoparticles, are one method for shielding RNA from extracellular ribonucleases, resulting in improved delivery efficiency and immunogenicity.^342–344^ Clinical studies have been initiated for several personalized cancer vaccines based on lipid nanoparticle-mRNA formulations.^177^ The lipid nanoparticle-formulated mRNA-4157 and mRNA-4650 vaccines are used alone in individuals with primary solid tumors or in combination with PD-1 inhibitor (NCT03313778, NCT03897881, NCT03480152).^338^ Advanced RNA-lipoplex formulations have been developed and explored as therapeutic cancer vaccines in several clinical studies owing to their advantage in systemic DC targeting and synchronized induction of highly potent adaptive and innate immune responses (NCT02410733, NCT02316457).^345,346^ Another point worth noting in mRNA vaccine delivery is the various oncology-related administration routes.^177^ Intravenous administration is preferable over intradermal or subcutaneous injection for mRNA-lipoplex vaccination, which induces a higher level of T cell responses in syngeneic tumor models.^345^ The route of administration mechanically determines the antagonistic effects of IFN on mRNA-lipoplex vaccines-induced T cell response. When mRNA-lipoplex vaccine is delivered subcutaneously, IFN signaling inhibits the antigen-specific T cell response; conversely, IFN increases T cell responses when administered intravenously.^345,347,348^ Intravenous injection has been widely used for the clinical administration of mRNA vaccines, which can deliver mRNA vaccine into direct intratumoral injection-inaccessible malignancies or those without reachable lymph nodes (NCT03897881, NCT03480152, NCT03908671, and NCT03948763).^177^ Altogether, neoantigen-based mRNA vaccines benefit from approaches that preserve their stability and improve the delivery efficiency.

In contrast to RNA and peptide vaccines, DNA vaccines are a multifunctional platform with numerous benefits, such as the ability to accommodate any sequence without affecting its stability or solubility, rapid industrial manufacturing at low cost, and easy storage without complicated cold-chain procedures. The DNA sequence encoding the predicted neoantigens is constructed into a suitable expression vector, which is amplified and purified in prokaryotic cells like *Escherichia coli*. Plasmid DNA is then introduced into cells or tissues via intramuscular or subcutaneous injection in combination with electroporation, where neoantigen is expressed to induce immune responses.^349^ DNA vaccines also offer a significant advantage in boosting immunity, including activation of humoral immunity via antigen-induced CD4^+^ and CD8^+^ T cell responses and stimulation of innate immune response by recognition of the double-stranded DNA structure.^350–353^ Rational selection of tumor-specific neoantigens can improve the immunogenicity of DNA vaccines by broadening immune responses and overcoming concerns, like antigen loss, modification and tolerance. A DNA vaccine based on polyepitopic neoantigens induces similar therapeutic anti-tumor responses achieved by peptide vaccines in mice bearing mammary tumors E0771 or 4T1.^308,350,354^ Combining a therapeutic DNA vaccine and anti-PD-1 therapy synergistically controls tumor growth in mice.^336,355^ An optimized polyepitope neoantigen DNA vaccine that encodes long epitopes linked with mutant ubiquitin also elicits strong neoantigen-specific immune responses in patients with pancreatic neuroendocrine tumors when paired with ICB therapy.^308^ There are numerous neoantigen-based DNA vaccine clinical trials being conducted for solid tumors, including TNBC, advanced small cell lung cancer, glioblastoma, pancreatic cancer, and pediatric recurrent brain tumor (Table 4).

Even though the personalized mRNA and DNA vaccines show less efficacy and success than ICBs and T cell therapies, tremendous improvements are still being made in the formulations and preparations of nucleic acid cancer vaccines, which will further accelerate the clinical application of neoantigen-based personalized nucleic acid vaccines in cancer patients.^156,334^

### Dendritic cell vaccines

APCs like DCs continuously present antigens to the immune system, making them an effective platform for delivering neoantigens. Autologous DCs can be isolated from patients and exposed to neoantigens, which are then injected back into the patient to elicit neoantigen-specific immune responses. Ex vivo loading of blood-isolated monocytes or hematopoietic progenitor cells with tumor neoantigens effectively improves the anti-tumor effects of neoantigen-based vaccines. Neoantigen-loaded DC vaccines can expand the antigenic breadth and clonal diversity of anti-tumor immunity.^9,112,356–361^ Several clinical trials are investigating the efficacy and safety of personalized neoantigen DC vaccines in solid tumors, such as melanoma, bladder cancer, colorectal cancer, esophageal cancer, breast cancer, ovarian cancer, pancreatic cancer, hepatocellular carcinoma, lung cancer, and gastric cancer (Table 4).

Neoantigens can be loaded to DCs by a variety of techniques, including pulses with the whole mRNA derived from autologous tumors, pulses with synthetic peptides and pulses with autologous whole tumor lysate (WTL), and fusion with tumor cells. The mRNA transfection is the simplest method for intracellular neoantigen production in DCs. Beyond introducing neoantigens, mRNA electroporation can also deliver functional proteins into the DCs, providing additional activation and maturation signals.^362^ The whole tumor mRNA-transfected DC vaccines induce T cell responses in vitro and improve the survival of immune responders with advanced melanoma (NCT01278940).^363^ The whole tumor mRNA-loaded DC vaccines also elicit neoantigen-specific T cell responses and exhibit safety in patients with various tumors, including melanoma, renal cancer, prostate cancer, uterine and ovarian cancer, colorectal cancer, pancreatic cancer, multiple myeloma and AML.^335^

Direct pulsing with synthetic peptides is another easy technique to load DCs with neoantigen-derived epitopes, which induces the necessary immune responses. This method requires the accurate identification and prediction of existing suitable epitopes in individuals, which are then synthesized into peptides or even full-length proteins to properly trigger an antigen presentation by the patient’s HLA repertoire on DCs.^362,364^ In several clinical trials, personalized neoantigen peptide-pulsed DCs have been tested against cancers, including melanoma, ovarian cancer, NSCLC and pancreatic cancers. DCs pulsed with synthetic long peptides and adjuvant Poly(I:C) broaden the breadth and diversity of neoantigen-specific T lymphocytes in melanoma. The t(2;13) translocation in 80% of alveolar rhabdomyosarcomas results in a PAX-FKHR fusion protein that is endogenously processed to generate a breakpoint epitope presented by HLA-B7. Stimulation of DCs with the SPQNSIRHNL fusion peptide derived from the PAX-FKHR neoantigen produced a specific CTL effect, resulting in lysis of rhabdomyosarcoma tumor cells.^365^ DCs pulsed with AR and ESFT fused neoantigen-specific breakpoint peptides, including EWS/FLI-1, EWS/FLI-2, PAX3/FKHR, and rhIL-2-treated autologous lymphocytes were reinfused to patients, and this regimen produced an immune response rate of 39% against the fusion breakpoint peptide.^100,366^ A personalized neoantigen peptide-pulsed autologous DC vaccine is also combined with chemotherapy or ICBs to treat patients with advanced lung cancer and pancreatic cancer (NCT05195619, NCT04627246, NCT02956551).

DCs pulsed with autologous WTL are safe and effective at inducing a broad anti-tumor immunity which have been extensively studied in various malignancies. In recurrent ovarian cancer patients, autologous DCs pulsed with oxidized WTL are well tolerated and elicit potent anti-tumor T cell responses. The vaccination amplifies T cell responses against neoepitopes originated from somatic mutations, including T cell clones against novel neoepitopes and clones with significantly higher avidity against known neoepitopes.^367–369^ Furthermore, neoantigens can be loaded into DCs by electrofusion technology, which fuses only the cytoplasm of two cell types without damaging the nucleus, thus maintaining the cellular function of these cells. In addition to expressing the tumor antigens, the fusion cells also enhance the co-stimulation ability of DCs.^315^ DC-tumor cell fusion vaccines have been tested in renal cancers, breast cancers, multiple myeloma and melanoma. In a subset of patients with renal cancer, the fusion cells induce tumor-specific immune responses and disease regression.^325,370–372^ Collectively, these preclinical and clinical studies have proven that neoantigen-based DC vaccines can elicit tumor-specific T cell responses, suggesting a feasible, safe, and effective immunotherapy for solid tumors.^373^

### Neoantigen-based adoptive cell therapies

Neoantigens with high immunogenicity, as described above, provide excellent targets for the ACT, which employs patients’ own naturally existing or genetically engineered anti-tumor lymphocytes. Neoantigen-based adoptive cell therapies, including TILs and genetically engineered immune cells with novel TCRs or CARs, are currently successfully used to treat multiple malignancies.^374^

### Adoptive transfer of TILs

CD8^+^ T lymphocytes have the capacity to identify and eradicate cancer cells, as discovered over 50 years ago.^375^ It has been demonstrated that adoptive transfer of in vitro expanded autologous TILs without genetic modifications can induce a full remission of certain human cancers. These TILs are taken from the patient, expanded under particular circumstances, and primed to increase their anti-cancer activity. Then, this cell product is reinfused back into the same patient, who have previous non-myeloablative lymphodepleting chemotherapy and subsequent cytokine therapy, like IL-2, thereby stimulating a potent anti-tumor immune response (Fig. 5).^376,377^ TILs enriched in specificity for neoantigens are preferable to unselected TILs at achieving complete and durable tumor regression. Compared to the low avidities of tumor antigen-specific TCRs, the majority of neoantigen-specific TCRs display significantly higher avidities, even towards cognate antigens expressed at relatively lower levels.^378^ Even a modest number of T lymphocytes with an affinity for scarcely tumor-specific neoantigens can be expanded for therapeutic application with the proper manufacturing process. Adoptive transfer of TILs enriched in neoantigen-targeted T cells is a promising treatment strategy, even for tumors with a low mutational burden.^379^

Neoantigen-reactive TILs mediate a remarkable regression of epithelial cancers, including advanced breast cancer, metastatic cholangiocarcinoma, colorectal cancer, melanoma, and cervical cancers.^28,380–385^ In the earliest prospective study of neoantigen-reactive T cells in epithelial cancers, metastatic cholangiocarcinoma patients with low TMB showed effective tumor regression lasting up to 35 months, offering the first concrete proof that neoantigen-targeted TILs can induce regression of metastatic epithelial cancer. Retrospective analysis of the infusion product has shown that the CD4^+^ T-helper 1 cells were reactive to an ERBB2IP mutation, suggesting a potential function of neoantigen-specific CD4^+^ T cells in the control of a metastatic epithelial cancer.^386^ TILs from individuals with metastatic gastrointestinal cancers have CD4^+^ and/or CD8^+^ T cells that recognize neoantigens resulting from somatic tumor mutations. Even though no common immunogenic epitopes are shared in these patients, a prevalent hotspot driver mutation KRAS-G12D in numerous patients can be targeted by CD8^+^ TILs.^271^ Similarly, in patients with metastatic colorectal cancer, KRAS-G12D mutant-targeted CD8^+^ TILs induce an efficient anti-tumor immune response against lung metastases that expressed HLA-C*08:02.^387^ The potential anti-tumor effect of neoantigen-reactive T cells has also been supported by retrospective investigations on the infusion of TIL products in patients with solid tumors. Patients with HPV16^+^ metastatic cervical squamous cell carcinoma have a full response to TILs that were initially selected based on their sensitivity to HPV antigens together with a high-dose of IL-2.^388^ Follow-up studies found that nearly 35% of the TILs could recognize the antigens resulting from tumor mutations compared to the 14% of the viral antigen-reactive TILs, indicating that the personalized neoantigen-reactive CD8^+^ T cells were responsible for tumor regression.^219^

TILs have been utilized to treat patients with metastatic malignancies who are refractory to current therapies, including chemotherapies, radiotherapies and anti–PD-1 therapies.^389–394^ Adoptive transfer of TILs targeting specific mutations in four genes, CTSB, CADPS2, KIAA0368, and SLC3A2, along with IL-2 and pembrolizumab results in a full durable regression of chemo-refractory HR^+^ metastatic breast cancer, which is still active at the last follow-up, 5.5 years after therapy.^377^ Patients with metastatic melanoma who are resistant to current therapies might achieve objective response rates of 50% to 70% with autologous TIL transfer and IL-2 after host lymphodepletion by total-body irradiation or chemotherapy.^395^ Patients who have metastatic NSCLC and are refractory to anti–PD-1 therapies showed a clinical response to immunotherapy combining the TILs, IL-2, and anti–PD-1 (NCT03215810, NCT04032847).^389^ Altogether, these studies have provided strong evidence that neoantigen-reactive T cells can improve the clinical outcome of epithelial cancers resistant to current therapies.

The frequency and breadth of TILs are key determinants of their therapeutic efficacy. The quantity and quality of tumor-reactive TILs are unambiguous variable across cancers with complex correlations with anti-tumor immune responses. For example, tumor-reactive TILs are limited to a small number of cells, as only about 10% of intratumoral CD8^+^ T cells can recognize autologous TSAs in ovarian and colorectal cancers, even no tumor-reactive TCRs have been found in some patients with the presence TILs.^396^ In contrast, neoantigen-reactive TILs have been detected in infusion products derived from nonresponding patients with metastatic breast cancer, gastrointestinal cancer, and NSCLC.^389,397,398^ Therefore, assessing the proportion of intratumoral T cell repertoires and their ability to recognize autologous tumors is critical for predicting the clinical activity of human cancer immunotherapies. Human CD8^+^ TILs can recognize a wide range of epitopes other than tumor antigens, such as antigens derived from viruses, forming bystander T cells that may infiltrate the tissue as effector cells. Neoantigen-specific TILs frequently exhibit stronger anti-tumor activity and tumor-specific expansion as compared to blood-emigrant bystander and regulatory TILs at various signatures and phenotypes.^399–403^ CD39, a marker of T cell reactivity to tumors and T cell exhaustion, can be used to identify the tumor-reactive T cells in a variety of malignancies. The bystander CD8^+^ TILs have overlapping characteristics with tumor-specific cells but lack CD39 expression and signs of persistent antigen stimulation at the tumor site.^396^ Furthermore, the frequency of CD39 expression in CD8^+^ TILs correlates with several important clinical parameters, such as the mutation burden and survival rate.^396,404^ Therefore, CD39 may be a promising indicator for evaluating the prognosis of cancer immunotherapy.^405^ The expression of CD39 could also be a viable biomarker for identification, isolation and expansion of tumor-reactive T cell populations in cancers.^404^ Using Cellular Indexing of Transcriptome and Epitopes by sequencing (CITE-seq) and TCR sequencing based on the signatures, such as CD39 and CXCL13 expression, neoantigen-reactive TCRs in NSCLC TILs can be identified with a success rate of 45% for CD8^+^ and 66% for CD4^+^ T cells.^406^ The immunomagnetic cell sorting of stem-like, self-renewable and tumor-specific TILs based on CD39 expression increases the median survival of mice by 60%.^407^ Collectively, optimizing the quality of the intratumoral TCR repertoire that is tumor-specific will improve the therapeutic potency of ACT.^396^

The intrinsic properties of the transferred T cells, including phenotype, avidity and persistent time, also influence the efficiency of neoantigen-directed ACT.^397^ High-dimensional analysis of TIL products identified two CD8^+^ T cell populations: one has a memory-progenitor CD39-negative stem-like phenotype (CD39^-^CD69^-^) and the other has a highly differentiated exhausted CD39-positive state (CD39^+^CD69^+^) TILs.^375,408^ The persistent exposure of TILs to antigens within the intratumoral microenvironment markedly shifted their phenotype towards an exhausted cell state (PD1^+^CD39^+^), accompanying by a progressive loss of CD8^+^ T cell activities and overexpression of inhibitory receptors like PD-1.^375,378^ It has been recently discovered that PD-1^+^CD8^+^ T cells retain a less differentiated subpopulation of stem-like TILs with ability of self-renewal, expansion, persistence, terminally differentiation and superior anti-tumor activity in vivo. These memory-like or progenitor-exhausted PD-1^+^CD8^+^ T cells serve as a source of terminally exhausted T cells that are capable of killing target cells.^375,378,409^ In contrast to the ACT non-responders, ACT responders have a reservoir of stem-like neoantigen-reactive TILs that expand prolifically and supply differentiated subsets, promoting T cell persistence and long-term tumor control.^375,408^ Consistent with their exhausted status, progenitor exhausted cells displayed inadequate enrichment for a central memory signature as opposed to an effector memory signature, relative to that of true central memory cells.^409^ When compared to T cells produced from an effector memory source, those from a central memory population show a stronger replicative potential in response to antigen and a longer in vivo persistence.^410^ The disentanglement of TIL exhaustion through isolating and expanding a desirable neoantigen-specific T cells with memory phenotype, engineering T cells to have stem-like properties, boosting the memory specificities outside the tumor with cancer vaccines, could pave the way for the creation of more effective T cell-based immunotherapies.

### Genetically engineered anti-tumor immune cells

Immune cells, including T cells, natural killer (NK) cells and macrophages, can be genetically modified in vitro to generate TCRs and CARs that redirect their specificity to neoantigens. These engineered immune cells circumvent the issues such as limited proportion of tumor antigen-reactive TILs.^411–415^ Since tumor neoantigens encoded by tumor-specific somatic mutations have emerged as primary antigenic targets of CD8^+^ and CD4^+^ T cells in ACT therapy without the toxicity of targeting normal tissues, rapid development of neoantigen-based immune cells holds promising effects for the treatment of solid tumors.^416^ A number of neoantigen-targeted TCR-T and CAR-T therapies are being actively investigated in early phase clinical studies, which show an intriguing therapeutic prospect (Table 4).^417^

### TCR-T cells

TCR-transduced T cells can target any surface or intracellular antigens. Several groups have proved the viability of an efficient approach from neoantigen identification to the engineering of the neoantigen-targeting cytotoxic TCR-T cells.^416,418–420^ When neoantigens are identified and predicted, neoepitope-specific T cells are isolated and their TCRs are sequenced. Candidate TCR sequences with known neoantigen reactivity can be introduced into T cells by transposon or CRISPR/Cas9 systems. These engineered cells expressing TCRs that are specific to neoantigens were infused into the patient after being verified for their tumor reactivity.^416^

The engineered high-avidity TCRs render CD8^+^ T cells specifically cytotoxic to neoantigen-containing tumors. The TCRs specifically targeting recurrent fusion genes CBFB-MYH11 confer CD8^+^ T cells antileukemic activity in vitro and in patient-derived murine xenograft (PDX) models with fusion gene-driven AML.^88,419,421^ Similarly, peripheral blood lymphocytes transduced with TCRs highly reactive to the mutated KRAS variants G12V and G12D could recognize multiple HLA-A*11:01^+^ pancreatic cell lines bearing the appropriate KRAS mutations in a xenograft model.^419,420^ The safety and efficacy of autologous T cells that have been engineered to express TCRs particularly targeting the HLA-A*11:01-presented public neoantigens, KRAS-G12V or G12D, are investigated in a clinical trial enrolling patients with advanced pancreatic cancer (NCT04146298, NCT05438667). Moreover, autologous T cells engineered with personalized neoantigen-specific TCRs are also being conducted in solid tumors, such as ovarian cancer, lung cancer, colorectal cancer, pancreatic cancer, cholangiocarcinoma and gynecologic cancer (NCT05292859, NCT05194735, NCT04520711).

In TCR-T therapy, replacing the endogenous TCR with a neoantigen-specific TCR (neoTCR) can precisely redirect the T cells to tumor cells with specific neoantigens presented by HLA. A recently developed non-viral precision genome editing technique can simultaneously knock-out the endogenous TCR or CAR genes and introduce a neoTCR or CAR, allowing a faster production of clinical-grade T cells.^422,423^ Based on this non-viral precision TCR replacement technology, a variety of T cell products with distinctly personalized neoTCRs for one patient are available to improve the anti-tumor effect. Three TCR-T cell products with unique personalized neoTCRs were administered to each of sixteen patients with refractory solid cancers, five of which had stable disease and the other 11 had disease progression as best response on therapy.^422^ Therefore, it is feasible and safe to create a broadly applicable, tumor-specific, and tailored T cell treatment for patients with solid malignancies based on this non-viral precision TCR replacement approach.

### CAR-T cells

CAR-T cell approaches have a substantial advantage over TCR-T cells since they do not rely on HLA expression and neoantigen presentation, the loss of which are commonly exploited by cancer cells for immune evasion. The engineered expression of CAR molecules, which contain an intracellular signaling and co-signaling domain and an extracellular antigen-binding domain, enable CAR-T cells to bind any cell surface protein once for which there is an antibody and then activate CAR-T cells independent of MHC.^424,425^ Early clinical trials using CD19-targeted CAR-T cells for the treatment of B-cell malignancies patients showed outstanding results, while CAR-T cells for the treatment of patients with solid cancer showed poor outcome because of the limited antigens.^424^ Tumor neoantigens have inspired creative solutions and given solid tumor patients hope for CAR-T therapy. The limited number of tumor-specific surface neoantigens that are suited for CAR-T can be overcome by integrating a single-chain variable fragment (scFv) that recognizes a neoantigenic pMHC complex on the tumor surface. CAR-T cells with an scFv that recognizes the oncogene nucleophosmin (NPM1c) epitope-HLA-A2 complex demonstrated strong cytotoxicity against NPM1c^+^HLA-A2^+^ leukemia cells and AML blasts with no or minimal on-target/off-tumor toxicity.^336,376,426^

CAR-T cells redirected at novel neoantigens are being tested in ongoing clinical trials in hematological and solid tumors.^424,427^ The most well-known example of neoantigen-based CAR-T therapy in solid tumors is neoantigens from EGFRvIII mutation, which are caused by in-frame deletion of a piece of the extracellular domain spontaneously in 30% of glioblastoma patients,^428^ making it a desirable target for CAR-T therapy. A CAR that can recognize the EGFRvIII neoantigen has been created as a part of a lentiviral vector and a truncated EGFR that lacks the ligand binding domain and cytoplasmic kinase domain is incorporated for in vivo tracking and ablation of CAR-T cells in necessary. Human EGFRvIII^+^ xenogeneic subcutaneous and orthotopic models showed that EGFRvIII-directed CAR-T cells could control tumor growth.^429^ The safety and effectiveness of autologous anti-EGFRvIII CAR-T cells are also tested in a pilot project in patients with recurrent glioblastoma (NCT02844062). However, only a small portion of tumor cells would be killed by targeting EGFRvIII due to the highly heterogeneous of glioblastoma.

Even if the antigens are imperfectly specific individually, a Boolean logic gate can be used in CAR-T cells to improve the specificity of tumor recognition by priming with tumor-specific neoantigens and boost the eradication efficiency of tumor cells by targeting antigens uniformly expressed by tumors. The T cells can generate CARs that target antigens universally expressed by tumors, like EphA2 and IL13R2, after being primed by a highly tumor-specific neoantigen, like EGFRvIII, and being trained to carry out complete tumor destruction. In addition, a synthetic Notch (synNotch)-regulated CAR activation maintains a significant proportion of T cells in a naive/stem cell memory state, leading to improved anti-tumor immunity. In immunodeficient animals bearing intracerebral PDXs with a heterogeneous expression of EGFRvIII, EGFRvIII synNotch-CAR-T cells outperformed conventional constitutively expressed CAR-T cells in terms of anti-tumor activity and T cell persistence without causing off-tumor damage. T cells engineered with prime-and-kill circuits induce CAR-driven cytotoxicity that is spatially limited only to the proximity of priming cells, preventing off-tumor killing in distant normal tissues that carry the killing antigen but lack the priming antigen.^231,430,431^

### CAR-NK

In addition to T cells, NK cells can also be engineered to express CARs. NK cells have the same capabilities as CD8^+^ cytotoxic T cells but they are not dependent on MHC-I -mediated tumor neoantigen presentation. As a result, the CAR-NK cells have the potential for immunotherapy against tumors with an extremely low mutational load and deficient neoantigen presentation. Arming NK cells with neoepitope-specific CARs remarkably improve their anti-tumor responses to NPM1-mutated AML without causing off-target toxicity.^432^ Moreover, NK cells further prime the DC maturation and neoantigen presentation via releasing GM-CSF, and recruit neoantigen-specific CCR5^+^CD8^+^ T cells by producing CCL5.^433^ Thus, the variety of cancer types amenable to immunotherapy increases as a result of modified NK cells.^7^

### Antibody-based therapy against neoantigens

Antibody therapies have been successfully used to treat cancers, such as the anti-PD1/PD-L1/CTLA4 antibody for ICBs. Compared to the conventional antibodies that are incapable of targeting intracellular proteins, TCR-mimic (TCRm) antibodies or mutation-associated neoantigens (MANA)-specific antibodies can recognize the intracellular neoantigens by focusing on pMHC complexes. TCRm antibodies have a greater affinity than TCRs, which has been shown to be essential for minimizing the on-target, off-tumor effects.^434–439^ These neoantigen-targeted antibodies are simple to transform into a variety of therapeutic formats, including full-length antibodies, antibody-drug conjugates (ADCs) and BsAbs. As mentioned above, TCRm antibody moieties can also be employed to drive specific activity for neoantigens by CAR-T therapy, which has proved remarkably effective in treating certain cancers.^440^ Moreover, these antibody-based immunological strategies have the potential to develop off-the-shelf products for any patient whose tumors exhibit the targeted public neoantigens.^300^

Phage display, yeast display and genetic platform are some of the technologies used to determine human TCRm antibodies with exquisite specificity for the neoantigen as presented on HLA. In order to identify scFvs specific for mutant pMHC complex, a phage or yeast display library encoding a vast number of scFv sequences was initially created. Using a competitive selection technique, clones specific for mutant peptides bound to predetermined HLA types were subsequently identified.^441,442^ A high-throughput genetic platform, PresentER, is comprised of minigenes that encode MHC-I peptide libraries. By assessing the reactivities of TCR-like therapeutic agents against vast libraries of MHC-I ligands, PresentER could be utilized to determine the on-and-off targets of T cells and TCRm antibodies.^443^ Combining structural analysis of a reagent with its corresponding pMHC complexes with library screening helps improve TCRm antibody specificity evaluations.^300^ According to a crystal structure, a human TCRm antibody called ESK1 attaches to Wilms tumor (WT1)-derived peptide/HLA-A*02:01 in a manner distinct from TCRs. The possible patient pool for ESK1 therapy can be expanded by using the structure to anticipate high-affinity binding of ESK1 with several different HLA-A*02 subtypes and potential off-target binding.^444^

Public neoantigens originating from recurrent driver mutations, including oncogenes and TSGs, provide shared targets that could benefit a substantial proportion of patients. The scFvs that target the public neoantigens coming from oncogene mutations, such as EGFR, KRAS, PIK3CA, and CTNNB1, have been identified and transformed into therapeutic formats.^300,441,445–447^ For example, one scFv specific for KRAS mutant-derived peptide and one for EGFR mutant-derived peptide has been identified by phage display. These scFvs recognize the peptides only in complexes with HLA, such as KRAS peptide/HLA-A2 or EGFR peptide/HLA-A3 complexes. The scFv specific for KRAS(G12V)-HLA-A2 is converted to a full-length antibody, which responds with mutant peptide-HLA complexes even when the peptide differs from the normal wild-type form by just one amino acid.^441^

In contrast to oncogenes, public neoantigens coming from recurrent mutations in TSGs are unable to trigger an immune response because they are either rendered inactive by non-recurrent mutations or produced at low levels due to nonsense-mediated RNA decay. The well-characterized TSG p53 is a special case due to the identification of TCRm antibodies that target the p53 pMHC complex. Due to MHC-binding restrictions, peptides containing mutant p53 sequences are uncommon; however, tumors expressing mutant p53 may have increased expression and the MHC molecule-mediated presentation of wild-type p53 peptide, which distinguishes the tumors with mutant p53 from healthy cells expressing wild-type p53.^448–451^ Therefore, a TCR-like antibody P1C1TM that is specific for the wild-type p53_125-134_ peptide in complex with the HLA-A24:02 (HLA-24) MHC allele can target tumors harboring mutant p53 and HLA-A24. This specificity for the p53 peptide/HLA-A24 complex enables P1C1TM as an antibody-drug conjugate to effectively deliver a cytotoxic payload to tumors with mutant p53, as demonstrated by the lethal effects of PNU-159682-P1C1TM restricted to mutant p53-expressing colorectal cancer cells in in vivo models.^451^

BsAbs can be employed to address the issue that the density of the mutant p53 pMHC complex on the cell surface was insufficient to recruit T lymphocytes to the tumor site. Bispecific T cell engager (BiTE) is a bsAb construct that provides an efficient and potent signal for T cell activation through simultaneously binding a neoantigen on tumor cells and a CD3 complex on T cells. Even when the neoantigen-MHC complex is expressed at low levels, the highly powerful bsAb is able to decisively reverse the undruggable reputation of p53.^317,440^ A peptide produced from the p53 missense mutant (R175H) can be presented by HLA-A*02:01 to form a mutant p53 pMHC complex at the cell surface, which serves as a natural TCR ligand to activate T cells. An H2 antibody fragment with enhanced affinity for the HLA-A*02:01-restricted p53 R175H neoantigen has been discovered by screening utilizing a large phage library. This TCRm antibody fragment was fused with a CD3-specific antibody fragment to create a bsAb that could improve the activation of T cells to recognize and destroy cancer cells and grafts in animal models expressing the p53 R175H pMHC complex.^440^

Dimeric T cell engaging bsAbs are also created based on human TCRm antibodies with exquisite specificity for the mutant LMP2A peptide-HLA-A*02:01 and mutant RAS peptide-HLA complexes. These bsAbs were effective in precisely activating T cells and killing target cancer cells that expressed endogenous, incredibly low quantities of the mutant neoantigens and cognate HLA alleles.^452,453^ In addition, bsAbs were also employed to target public neoantigens originating from dysregulated PTM in malignancies. BsAbs engaging CD3 with TCRm specific for a pIRS2-derived phosphopeptide in complex with HLA-A*02:01 were capable of killing tumor cells in a pIRS2- and HLA-A*02:01-restricted manner.^189^ Alternatively, soluble structures guided by monoclonal TCR moieties specific for tumor neoantigens can also be coupled to an anti-CD3 antibody component to generate a group of bispecific molecules, known as immune-mobilizing monoclonal TCRs against cancer (ImmTACs). ImmTACs get over the biophysical constraints that prevent TCR-based immunotherapeutic methods in the past and might make it possible to target any cell based on its proteomic traits. Cancer cells with extraordinarily low surface epitope concentrations were successfully killed by T lymphocytes that had been guided by ImmTACs.^300,454,455^

As a result, TCRm antibodies-based strategy could be used to target neoantigens originating from mutations in both oncogenes and TSGs that are challenging to eradicate using traditional methods, enabling the development of more targeted anti-cancer therapies.^452,456–459^ Given TCR mimic antibodies have a much better affinity to peptide-HLA molecules than natural TCRs. To prevent cross-reactivity or binding of the HLA component unrelated to the given peptide, TCR mimic antibodies must be properly screened. Similar to designed TCRs, cross-reactivity can be prevented by using negative selection against off-target peptides.^426,441,447^ At least one instance of synthetic reagents has been developed that exhibits lower cross-reactivity than equivalent natural receptors.^460^

### Combinational therapies

**Fig. 6:**
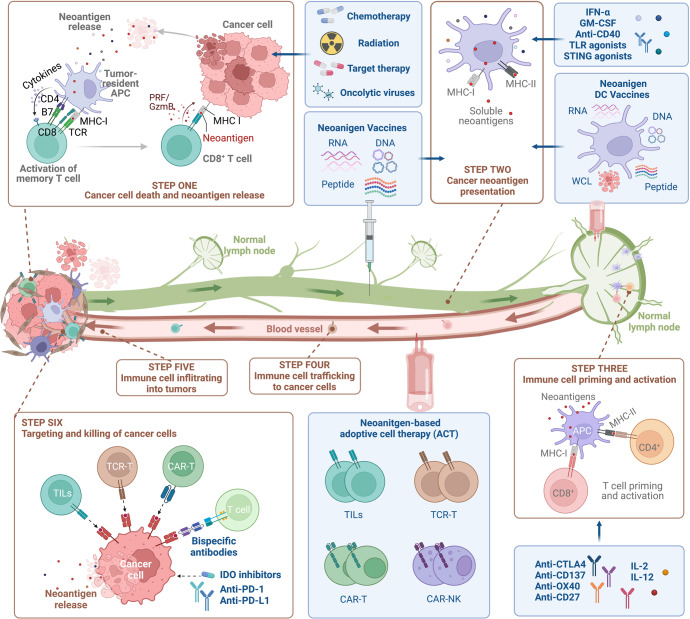
Combinational neoantigens-based anti-tumor strategies. The “Cancer-Immunity Cycle” refers to the sequential events that must be initiated, proceeded, and expanded to achieve an anti-cancer immune response, resulting in the efficient eradication of cancer cells. Briefly, neoantigens generated by oncogenesis are released and captured by DCs (step 1). DCs convey the collected neoantigens on MHC-I and MHC-II molecules to T cells (step 2), resulting in priming and activation of effector T cell responses against cancer-specific neoantigens (step 3). Subsequently, activated effector T cells migrate to (step 4) and infiltrate into (step 5) the tumor bed, where they recognize and finally destroy their target cancer cells (step 6). The death of cancer cells produces additional tumor-associated neoantigens (step 1 once more), which broadens and intensifies the immune response in subsequent cycles. Therefore, cancer immunotherapies have been designed to reinitiate or amplify a self-sustaining cycle of cancer immunity. Multiple immunotherapies have been developed to target the rate-limiting steps in “Cancer-Immunity Cycle”, including enhancing the neoantigen release by chemotherapy, radiation therapy and oncolytic virus, increasing the quantity and quality of tumor-reactive T cells through cancer vaccine and ACTs, and boosting the infiltration and cytotoxicity efficacy of immune cells via checkpoints inhibitors

The therapeutic efficacy of a single immunotherapy for patients with advanced cancer is inadequate due to the heterogeneity of the neoantigen landscape and the continually evolving cancer immune evasion mechanisms. Combining several immunotherapies can improve the efficacy against cancers by simultaneously targeting various stages of the cancer-immunity cycle, including antigen release and presentation, immune cell priming and activation, immune cell trafficking and infiltration into tumors, and recognition and killing of cancer cells.^7,461^ Another strategy is combining therapies with different mechanisms of action to overcome the resistance induced by tumor heterogeneity. All targeted cancer cells must have the same pattern of neoantigen expression and presentation, otherwise, a resistance clone without the predicted neoantigens can survival and confer a clonal growth advantage. Therefore, precision immunotherapy can be combined with conventional treatments like radiotherapy and chemotherapy that kills cancer cells independent of the neoantigens, achieving a more prominent and durable therapeutic effect (Fig. 6).

### Neoantigen-based immunotherapies and ICBs

Checkpoint inhibitor-based immunotherapy has achieved prolonged anti-tumor effects in several malignancies, including renal cell carcinoma, NSCLC and melanoma. Patients, however, do not react to ICB therapy in the absence of tumor-specific effector T cells.^245^ Moreover, ICB therapy only affects one or two phases of the anti-cancer immunity pathways, such as anti-CTLA4 antibodies regulate the immune cell priming and activation, while anti-PD-1/PD-L1 antibodies focus on the final negative regulation of T effector cells. Therefore, only a small percentage of patients have anti-tumor response with a single agent. The neoantigen load and intratumor heterogeneity can be predictive biomarkers for the ICB response.^38^ It is reasonable to suspect that more effective anti-tumor response would be achieved by combining ICBs with neoantigen-based immunotherapy approaches that boost the tumor-reactive T cells.^8^ ICBs enhance specific T cell responses by targeting neoantigens, including PRKDC, EVI2B and S100A9, in a relapsed multiple myeloma patient.^41^ Compared to monotherapy, the neoantigen vaccine (PancVAX) in combination with two checkpoint modulators, such as anti-PD-1 and agonist OX40 antibodies, causes an improved and more substantial tumor regression.^462^ For patients with solid tumors who are unresponsive to, or relapsed following anti-PD-1 therapy, mRNA-based neoantigen vaccines, such as mRNA-4157, mRNA-5671, and BNT122, are used together with immune checkpoint inhibitors in multiple clinical trials (Table 4).^177^ Frequently, immunosuppressant regulators, such as PD-1, PD-L1, CTLA-4, and TIM3, are upregulated by neoantigen vaccines.^245,433^ ICBs could mitigate this negative effect of neoantigen vaccinations, leading to fast and long-lasting CD8^+^ T cell control of malignancies.^177,433^ Therefore, the combination of neoantigen vaccines and ICBs can achieve a better expected effect of anti-tumor immune response.^245^

The anti-tumor efficacy of CTLs, including those specific for mutation-associated neoantigens, can be further boosted by ICB therapy. TILs often exist in small quantities within a tumor and demonstrate an irreversible hypo-responsiveness as a result of the suppressive microenvironment. Therefore, most cancer patients are not eligible for TIL therapy.^389,421^ Patients with immunotherapy response to PD-1 inhibitors have a high proportion of TILs, indicating that ICBs can promote the infiltration of neoantigen-reactive lymphocytes into tumors.^376,463^ Blocking the PD-1 inhibitory signals induce the expansion of PD-1^+^ CD8^+^ T cells, resulting in a transient elevated cycling PD-1^+^ CD8^+^ T cells and an increasing amount of effector T lymphocytes at the tumor site.^376,400,464–467^ In addition, ICBs can reinvigorate the exhausted neoantigen-specific T cells via overcoming the suppressive microenvironment. Persistent exposure to TSAs promotes the exhaustion of CD8^+^ T cells, which characteristically expressed high levels of PD-1 and CD39.^465,466^ The intratumoral CD8^+^ T cells with high PD-1 expression show an intrinsically high capacity for tumor recognition.^468^ Given the potent activation of CD39^+^CD8^+^ T cells by high-affinity neoantigens, patients with hepatocellular carcinoma in high-affinity neoantigens-high group benefited more from anti-PD-1 therapy than high-affinity neoantigen-low group.^123^

### Combinations of neoantigen vaccine and ACT

Combinations of neoantigen vaccination and ACT have also been utilized successfully to boost clinical efficacy in tumor treatment.^245^ Recent exciting findings showed that vaccination can increase the amount of neoantigen-reactive T cells in circulation, possibly by boosting better outgrowth of T lymphocytes. Alternatively, the vaccines can induce de novo T cell responses that overcome the insufficient recognition of neoepitope by T cells due to inadequate cross-presentation of a neoantigen by tumor cells. In addition, vaccines can be made to shield neoantigen-reactive T cells from immune checkpoint signaling or FasL-mediated apoptosis, allowing T cells to infiltrate the immunosuppressive tumor microenvironment (TME) and durably reduce epithelial malignancies. In order to increase the clinical efficacy of subsequent ACT therapy, vaccinations may be utilized to prime the patient’s neoantigen-reactive TILs or PBMCs before in vitro T cell culture. This could result in the induction of a known memory T cell response.^374^

Vaccine is also used to enhance the efficacy of CAR-T therapy to eliminate solid tumors. A booster vaccine for CAR-T cells has been designed, in which the peptide neoantigens can be trafficked to lymph nodes and subsequently decorated the membrane of resident APCs by their albumin-binding phospholipid-polymers. Vaccine-boosting donor cells enhance CAR-T function in solid tumors through their chimeric receptor directly in vivo. This amph-ligand vaccine can significantly elicit the amplification and intratumoral infiltration of EGFRvIII-specific CAR-T cells compared to CAR-T cell delivery alone. This vaccine strategy safely expands CAR-T cells in vivo and boosts their function and anti-tumor activity in multiple models of solid tumors, showing the significant promise of neoantigen vaccine and CAR-T combinatorial therapy.^245,469–471^

### Neoantigen-based immunotherapies and conventional therapies

The majority of chemotherapeutic agents and radiation therapy were designed based on their direct cytotoxic effects without considering their impact on immune system. The genomic damage and altered gene transcription during these conventional therapies can promote the production of tumor-specific neoantigens, hence exhibiting potential of stimulating the anti-tumor immune response. Therefore, several FDA-approved combination therapies using conventional therapy together with immunotherapy have been developed.^7^

Chemotherapy and radiotherapy can be used to increase the release of tumor-specific neoantigens, circumventing issues such as an insufficient number of neoantigens to stimulate T cell response. In a patient with metastatic NSCLC who has completed response to the combined CTLA4 blockade and radiotherapy, neoantigenic mutation in KPNA2 is upregulated by radiation. Peptides derived from mutant KPNA2 trigger neoantigen-reactive CD8^+^ T cells and induce IFNγ production, which may trigger antigen spread.^472,473^ In addition, radiation can enhance the levels of existing peptide presentation by increasing the surface expression of MHC-I on tumor cells. Though expanding intracellular neoantigen pools and increasing the MHC-I-dependent presentation, radiation would promote cell killing by neoantigen-specific CD8^+^ T cells.^472,474,475^ In a poorly immunogenic mouse model of TNBC, radiotherapy increases the expression of genes with immunogenic mutations. The neoantigen vaccines based on the immunogenic mutations induced by radiotherapy elicit CD8^+^ and CD4^+^ T cells that improved the therapeutic efficacy of radiotherapy.^476^ Notably, highly subclonal neoantigens induced by radiation, which might be worsened by DNA-damage response (DDR) inhibitors, would interfere with the production of T lymphocytes against clonal tumor neoantigens. Additional investigations on the formation of subclonal neoantigens, as well as a thorough investigation of combined radiation, DDR inhibitors, and neoantigen-based therapies are needed to address these concerns.^472^

During the chemotherapy and targeted therapy, the tumor cells often occur new mutations, including reversion mutation, contributing to drug resistance. Many reversions are predicted to encode tumor-specific neoantigens, offering a potential strategy for combating resistance with CAR-T cell therapies, immune checkpoint inhibitors or anti-cancer vaccines. Reversion mutations in breast cancer-related genes are just one example that occurs during clinical platinum and PARP inhibitor resistance.^477^ The amount and functional activity of neoantigen-specific T lymphocytes can also be increased by administering a tumor vaccination followed by pretreatment with cyclophosphamide (CTX) and other drugs.^177^ Together, these studies show proof-of-principle that conventional treatments can enhance tumor control when used in conjunction with immune therapies based on neoantigens.

## Challenges and opportunities for clinical application

Despite the success in hematological malignancies and solid tumors mentioned previously, neoantigen-based immunotherapies have only shown objective efficacy in a small number of well-documented patient responses. Consequently, considerable improvements are required to improve clinical results, including increasing the accuracy of neoantigen prediction, overcoming immune evasion, and optimizing the streamlining of the production process. This section focuses on the barriers that must be surmounted to enable potent immune response specifically based on tumor-specific neoantigens and the possible solutions for offering a safe and effective therapy for solid tumors.

### Limited accuracy of neoantigen prediction

The widespread application of personalized immunotherapies has been constrained by the limited discovery of targetable cancer neoantigens due to the heterogeneity of mutational burdens and significantly distinct neoantigen presentation among various tumor types.^81^ Only 10% of non-synonymous tumor cell mutations can produce mutant peptides with high MHC affinity, and only 1% of the MHC-binding peptides are recognized by patient T cells. Theoretically, the higher the TMB, the greater the number of neoantigen-specific T cells in the tumor can be detected, resulting in a greater immunotherapy response rate. Nevertheless, low TMB can produce neoantigen-reactive lymphocytes in hematological malignancies and certain epithelial cancers, such as gastrointestinal cancers.^10,240,478^ The insufficient neoantigen density in malignancies with low TMB, such as AML and pediatric brain cancers, requires greater powerful strategies for the accurate identification of immunogenic neoepitopes that can be detected by CD8^+^ T cells.^479,480^ High-throughput technologies enable systematic assessment of suitable neoantigens for immunotherapies, overcoming the limited neoantigens caused by low TMB.^333^ For example, a proteogenomic method that integrates NGS and MS data supports the development of highly target-specific, autologous, personalized neoantigen immunotherapy, especially for tumors with low TMB.^479,481,482^

The prediction of neoantigens is also constrained by genetic heterogeneity, particularly the diverse somatic mutations in distinct cancer types, in different individuals and even within tumor subclone cells. A major cause of genetic heterogeneity in cancer is genomic instability, which is dynamically altered in distinct tumors and different stages. For example, TNBC patients have a higher load of frameshift and mutation-associated neoantigens (MANA) and a higher response rate to immunotherapy compared with patients with other invasive breast cancer subtypes. Furthermore, BRCA-1-mutated TNBC has an even higher mutational load.^53,483–485^ Therefore, identification and prediction of neoantigens should be conducted uniquely for individuals with specific cancer.^245^ Additional problems may develop depending on how the tumor sample from a patient is obtained for neoantigen identification. Recent technologies enable the investigation of the genomes and transcriptomes of single tumor samples taken at specified time points; however, this does not disclose heterogeneous mutations occurring in different lesions across a patient. The diversity of neoantigen-specific T cells present in a patient may not be fully captured by a single excised lesion due to restricted T lymphocyte infiltration, which constrains the TCR repertoire that may be established for therapy.^245^ Furthermore, mutational heterogeneity within tumors contributes an additional degree of intricacy for neoantigen prediction. The genome of tumor cells undergoes extensive generation, cloning, alteration and loss of mutations. Thus, tumor clone cells that do not respond to the neoantigen-specific T cells may exist, which may outgrow other clones due to a selection advantage, thereby restricting clinical benefit.^40^

### Escape from immunological surveillance

A significant barrier to eliminating cancer is immune evasion, particularly for anti-cancer immunotherapies. Tumors can evade neoantigen-based immunotherapies through a number of mechanisms, including the loss of neoantigens, modification of antigen peptide presentation, and immunosuppressive TME.

### Loss of neoantigens

The loss of tumor-specific neoantigens may be a significant immune escape strategy for tumors, especially if many neoantigens are by-products of tumorigenesis and do not have a critical function in tumor cell survival. The depletion of neoantigens can also present as a refractory mechanism to anti-tumor immunity, limiting the application of individualized neoantigen-specific immunotherapy. Neoantigen depletion can be induced by multiple pathways, such as copy number loss, transcriptional repression, epigenetic silencing and post-translational mechanisms. In a cohort of early-stage NSCLC tumors, 48.9% (43/88) showed evidence of neoantigen loss due to subclonal copy number events. The mutant genes encoding non-expressed neoantigens have enriched hypermethylation at their promoter regions compared with the wild-type parental genes in other purity/ploidy matched samples.^486^ In addition, tumors can alter the presentation of neoantigens via modulating protein turnover. Mutant proteins are more likely to misfold and degrade quickly through the proteasome, resulting in elevated antigen presentation. Molecular chaperone HSP90, however, can be used by tumors to stabilize altered proteins, preventing them from entering the antigen presentation pathway.^487,488^ Neoantigens that exclusively existed in specific tumor cell subpopulations can also be lost as a result of the CD8^+^ T cell-mediated eradication of the entire subclonal cell population. Many of the deleted mutations are identified by patients' T cells and neoantigen-encoding genes are unlikely to be produced in tumors with extensive immune cell infiltration, suggesting that neoantigen-expressing tumor subclones may be preferentially removed by the immune system.^487,489^ Furthermore, neoantigen loss through the deletion of chromosomal regions or the elimination of tumor subclones can lead to acquired resistance to immunotherapies such as ICBs.^489^ Therefore, to compensate for the loss of targetable neoantigens during immunotherapy, personalized neoantigen-specific immunotherapy should target multiple neoantigens, therefore expanding the scope of neoantigen reactivity.^487^

### Disrupted presentation of neoantigen peptides

Tumors may evolve mutations that change not just neoantigen expression but also HLA heterozygosity and MHC stability in response to anti-tumor immune pressure. These changes impede neoantigen processing and presentation, hence inhibiting T cell recognition and tumor killing.^316,490^ The tumors may be able to avoid recognition by adoptively transferred T lymphocytes if there are mutations in key antigen presentation genes like β2M or a lack of HLA allele heterozygosity.^316,380^ For example, all seven lung metastases from a colorectal cancer patient regressed after receiving an infusion of TILs containing four unique T cell clonotypes that target KRAS-G12D. However, an evaluation nine months after treatment found that one of these lesions had advanced progress. Further analysis of this excised lesion discovered that the chromosome 6 haplotype responsible for the HLA-C*08:02 MHC-I molecule had been deleted, contributing to the tumor immune evasion.^387^ A second verified mechanism for epitope loss has been found as the downregulation of MHC molecules in tumor cells owing to aberrant transcription, translation or protein stability events.^491,492^ In multiple myeloma cell lines, higher levels of splicing factor expression are associated with lower levels of MHC-II activity, while spliceosome inhibition improved MHC-II activity, suggesting that abnormal alternative splicing is responsible for the loss of MHC-II.^123^ Moreover, autophagy-dependent degradation causes the lower expression of MHC-I in pancreatic ductal adenocarcinomas that are resistant to ICB treatment. Inhibiting autophagy improves antigen presentation and anti-tumor T cell responses, slowing tumor growth in syngeneic host mice.^493^ Due to the decreased neoantigen presentation, these mechanisms together may help to partially explain why higher neoantigen loads in some cancers were not linked to better prognostic outcomes. Based on these findings, ICB therapy for cancers may be more effective if MHC-I presentation is activated using splicing inhibitors or autophagy inhibitors.^123,493^

### The immunosuppressive TME

Loss of neoantigens and insufficient presentation are only two of the many immune evasion mechanisms possessed by tumor cells. The recognition of neoantigens and activation of T cells can also be compromised by immunosuppressive TME processes, including the suppression of immunological checkpoints, the immunosuppressive effects of various TME cells, and the release of ions or proteins from within tumor cells following necrosis.^245^ The immunosuppressive checkpoint ligand molecules like PD-L1 and CTLA-4, which can restrict T cell growth and function biologically, are often upregulated in tumor cells during immunotherapeutic treatment.^245,433,491^ ICBs are combined with neoantigen vaccines to prevent immune escape.^245^

Inducible neoantigen expression, combinatorial CARs, and leveraging epitope spreading are compensatory strategies that may be used to address the immune escape of tumor cells.^494^ The MHC-I immunopeptidomes can be extended by splicing-derived neoepitopes that result from defective interactions of splicing complex with RNA, improper degradation of the accessory splicing factors, or aberrant splicing factor PTMs. The considerable number of highly immunogenic neoantigens created by pharmacologically altered splicing enhances tumor immunogenicity and improves the immune response to ICB treatment in mouse model.^495^ These findings open the exciting possibility of employing immunotherapy, which was until now only effective in diseases with greater mutation burdens like melanoma, to treat cancers that are resistant to current treatment.

### Insufficient production of neoantigen-specific T cells

The immunotherapies, including vaccination, adoptive transfer of tumor-reactive TILs and TCR-T cell therapy, as well as ICBs, all rely on neoantigen-specific T cells. Thus, streamlining the sufficient production of tumor-reactive T cells from cancer patients or healthy donors should accelerate the application of neoantigen-based therapies, including broadening the TCR repertoire, enhancing the neoantigen presentation and the proper expansion of T cells.

### Limited neoantigen-reactive T cell repertoire

TILs include a large number of neoantigen-reactive T cells, making them a valuable source of T lymphocytes for ACTs.^380^ However, the implementation of TILs treatment will be hampered by the scarcity of fresh tumor samples and cold tumors with low TILs.^389,421^ First, the acquisition of TILs requires invasive surgery to remove a resectable lesion, which enables only some patients to be suitable. Second, in cold tumors, the suppressive TME may reduce the efficacy and quantity of TIL-derived neoantigen-specific T cells.^379,389,421^ Therefore, the majority of cancer patients are ineligible for TIL therapy due to insufficient TCR repertoire.^379^ Efforts are currently being made to develop efficient strategies for the isolation and rapid expansion of neoantigen-specific T cells, which could benefit neoantigen-based ACTs. Utilizing particular cell-surface markers such as CD39, immunomagnetic cell sorting can efficiently detect and separate potent tumor-specific TILs with self-renewing ability from solid tumors, enabling the long-term effectiveness of ACTs.^407^

The easily accessible peripheral blood could be a suitable source for generating large amounts of neoantigen-reactive T cells for ACTs.^379^ Both circulating neoantigen-specific T cells and peripheral blood lymphocytes modified to express neoantigen-specific TCRs recognize and kill autologous malignancies.^467,496,497^ There are several clinical trials utilizing neoantigen-reactive T cells generated from peripheral blood to treat patients with epithelial cancer (NCT02959905, NCT05020119, and NCT04596033).^498^ Nonetheless, these T cells are significantly scarce and need to be precisely isolated by identified neoantigens or stimulated in vitro to proliferate to detectable levels.^374^ Cancer patient-derived PBMCs can be stimulated in vitro with mutant peptides to enrich neoantigen-reactive T lymphocytes. However, it should be highlighted that the substantial in vitro expansion can both further differentiate T cells and expand falsely positive neoantigen-reactive T cells.^499,500^ Moreover, the tumor-reactive TCR repertoire of PBMC-derived T cells can be expanded by transducing TCRs specific for tumor neoantigens of each patient.^374^ In addition, numerous neoantigen-reactive TCRs can be simultaneously transduced into the PBMC of a patient to boost the immune response. Based on these advantages, minimally cultured T cells with individualized TCRs will have better cytolytic capacity than exhausted and senescent TILs. Therefore, future studies improving neoantigen-reactive T lymphocytes generated from peripheral blood will benefit for their clinical applications.

The efficacy of ACTs can be restricted when tumor-reactive T cells are weakly persistent and terminally differentiated effector cells.^501–503^ Naive and very early memory T cells with stem cell-like properties can be produced using induced pluripotent stem cell (iPSC) technology.^504^ The functionally regenerated CTLs produced from iPSC that are specific for the EWS/FLI1 fusion gene-derived neoantigen elicit an anti-tumor immune response in both cultured EWS/FLI1^+^ sarcoma cells and Ewing sarcoma xenograft mouse model.^505^ iPSC-derived immature T cell lineages can now be further differentiated into neoantigen-specific T cells based on the unique three-dimensional thymic organ culture system that faithfully recapitulates the disease in vitro. The ability of iPSC-derived thymic emigrants to express heterodimeric αβ co-receptor of T cells can be maintained by imitating the thymus in vitro.^506^ Another benefit is that iPSCs can be established from a single neoantigen-specific CTL clone and re-differentiated into a large number of CTLs.^505^ In the near future, iPSCs derived from a single CTL clone will be employed to generate a sufficient number of neoantigen-specific TCR-T cells that preserve a naive-like condition and contain the TCRs in its endogenous state, significantly boosting neoantigen-based immunotherapies.

### Neoepitope presentation

Presently, mature DCs or EBV-transformed B cell lines that are pulsed with peptide or transfected with TMGs for APCs are used to present antigens to T cells.^374^ Neoantigen-expressing mRNA-transfected DCs are also employed to prime the autologous naive CD8^+^ T lymphocytes in healthy donors, who are not exposed to the immunosuppressive milieu of tumor hosts.^101^ However, TCRs triggered by APCs pulsed with mRNA-encoding or synthetic peptides might not recognize tumor cells that present antigens endogenously.^507^ Directly detecting the neoantigens presented by tumor cells may be the most efficient method to establish neoantigen-reactive T cells, ensuring they can recognize the neoepitope in vivo. Tumor single-cell suspension, PDXs in highly immunodeficient mice and three-dimensional patient-derived tumor organoid cultures can be used for the recognition. Notably, PDXs and tumor organoids exhibit more typical features in neoantigen processing and presentation in contrast to the synthetic peptides or TMGs presented by DCs or B cells. PDXs maintain the endogenous expression, natural processing, and presentation of neoepitopes.^508^ When little tumor biopsies are available, PDX tumors can be employed to steadily extend the authentic peptidomes, creating possibilities for identifying neoantigens and sufficiently presenting neoepitopes to T cells.^509^ The tumor organoid cultures that extend the in vitro capacity of patient tumor cells are another alternative for autologous neoepitope presentation. A unique preclinical therapeutic model was created using tumor organoid-T cell co-culture systems to precisely measure each patient’s sensitivity to various immunotherapies. This approach was able to assess the effectiveness of cellular immunotherapies in vitro while also preserving the heterogeneity and microenvironment of tumors. In addition, co-culturing autologous PBMCs with tumor organoids produced personalized tumor-reactive CD8^+^ T lymphocytes that significantly reduced the growth of tumors.^405,510^ Although further improvements are required to conserve TME, including myeloid and stromal components, the organoid model may offer a better in vitro opportunity for neoantigen presentation and recognition by T cells.

### Improved expansion approaches for tumor-reactive T cells

A significant mechanism of immunotherapy resistance is the death of tumor-reactive T lymphocytes. Since the finding that the expression of Fas-ligand (FasL) in melanoma cells caused TIL mortality, the role of the Fas/Fas-ligand pathway in establishing tumoral immune resistance has been debated for several decades.^511^ T cells separated from cancer patients express more Fas than healthy donors. Moreover, most cancer cells and intratumoral vascular endothelial cells express high levels of Fas ligand (FasL), which is related to a lack of CD8^+^ infiltration.^374,512^ Fas-FasL interactions in the TME during ACT may lead to T cell death. Therapies to control the Fas-mediated apoptosis and differentiation may be helpful to generate the appropriate cell products for efficient ACT. The mutant Fas that failed to bind FADD are used as a dominant negative receptor (DNR) to prevent FasL-mediated apoptosis in Fas-competent T cells.^380^ The Fas DNR can be transduced together with a neoantigen-reactive TCR or CAR into T cells, which can be further enriched using a magnetic bead of the introduced TCR or Fas. These Fas DNR-engineered TCR-T or CAR-T cells showed improved persistent anti-tumor activity against established solid and hematologic malignancies.^380^ Other strategies involve the systemic administration of Fas-Fc or anti-FasL to neutralize FasL. These FasL-neutralizing methods may reduce the death of TILs, enhance the tumoral infiltration of CD8^+^ T cells, and improve the persistence and activity of T cells at the tumor site.^513^ Notably, when administered in concert with ACT, these cell-extrinsic reagents may impair the capacity of T cells to use Fas/FasL signaling to cause cytolysis in tumor cells.

### Determination and monitoring of neoantigen-specific T cell responses

Reliable immune monitoring will be essential to assess whether neoantigen-based immunotherapy achieves its expected immunologic effects and to expand the immunologically effective candidates to larger and suitable patient subsets.^514^ The tumor-reactive T cell responses are crucial for anti-tumor efficacy of various therapies, including cancer vaccines, ACTs, bsAbs and ICBs. The quantity and quality of tumor-reactive T cells can be measured and tracked in order to anticipate how well cancer immunotherapy will work.^378,515,516^ A multiparameter phenotypic and functional readout of T cell reactivity will be likely necessary in the absence of extensive evaluation using several types of T cells and APCs.^380^ Numerous effective markers, including CD39, PD-1, TIM-3, OX40, 4-1BB, IFNγ, and TNFα, can be used to determine the proportion of neoantigen-reactive T cells in infusion products and their capacity to recognize autologous tumors. CCR5 and CXCL13 can also be used as T cell-intrinsic indicators for CPI sensitivity. In addition, the neoepitope-specific CD8^+^ T cells in the blood can be used to identify the ongoing anti-tumor immune response at the tumor location. Blood neoepitope-specific T cells analyzed on a single-cell level revealed substantial clonal T-cell expansions with different effector transcription patterns, which are also present in the corresponding malignancies, indicating the recognition and destruction of tumor cells.^401^ It should be noted that the level of circulating tumor DNA (ctDNA), a proxy for tumor burden, can be utilized to dynamically detect the mutations that generate neoantigens. The ctDNA sequencing-base method can also monitor the neoantigen evolution during ICB treatment, thereby guiding personalized immunotherapy.^517^

The function and specificity of T cells can also be understood through examination of cellular states, which can also serve as a predictor for how well neoantigen-directed ACT would work. A reservoir of stem-like neoantigen-reactive TILs, such as CD8^+^ cells expressing activation or exhaustion markers (PD-1, TIM-3, and LAG-3), expand prolifically and supply differentiated subsets promotes T cell persistence and long-term tumor control, thereby strengthening the anti-tumor response.^518,519^ With high-throughput transcriptomic and TCR sequencing, neoantigen-specific TCR clonotypes dysfunctional characteristics were utilized to detect anti-tumor TCRs with minimal TIL material. Signatures of neoantigen-specific TCR clonotypes characterize the landscape of TILs across tumors, allowing TCR prediction based solely on TIL transcriptomic states for neoantigen-based cancer immunotherapy.^520^ These signatures may provide a degree of clonality predicting clinical response to various immunotherapies by identifying anti-cancer TCRs in the blood and, more crucially, in the tumor without the need for functional screening of putative neoantigens.^378,516^

## Conclusion and perspective

In summary, neoantigens play a pivotal role in cancer immunotherapies, including cancer vaccines, ACTs, antibody-based therapies, and ICBs. This review summarizes the compelling evidence indicating the therapeutic strategies by targeting these cancer-specific neoantigens without normal tissue destruction and provides a strong rationale that supports the relevance of neoantigens in clinically successful immunotherapies. Numerous initiatives are being made to develop personalized or off-the-shelf anti-cancer medicines based on neoantigens. Nevertheless, experimental and theoretical improvements are required to address the time and economic issues for advanced personalized neoantigen-based immunotherapies, including efficient recruitment of patients, optimizing sequencing technology and neoantigen prediction algorithms, and taking off-the-shelf therapies against public neoantigens.^15,245,302,309^

Effective patient recruitment is essential for neoantigen-based immunotherapy. First, early resection may improve clinical outcomes by giving clinicians more time to carefully design, produce and test neoantigen-based therapeutic medicines. Second, early resection makes it easier to select patients who might be eligible for the off-the-shelf therapies, including vaccines, TCRs or TCRm antibodies that target well-characterized cancer driver mutations. Third, given cancer treatment, including chemotherapy, radiation therapy and ICBs will stimulate T cells to become excessively differentiated, isolating autologous T cells or TILs as soon as a patient is diagnosed with cancer may enable the collection of the highest-quality and least differentiated T cells from patients.^409^ In addition, early patient enrolment in neoantigen-based therapies enable effective infusion of superior neoantigen-based cellular products and ameliorate the severity of co-morbidities caused by advanced metastatic cancer clones.

The precise identification of immunogenic neoantigens and their cognate TCRs is the most crucial and rate-limiting step in the creation of personalized cancer immunotherapies.^101^ Immunogenic neoantigens can be identified by both immunogenomic approach that builds virtual peptidomes using in silico algorithm based on NGS data and immunopeptidomic approach that uses MS to analyze the MHC-loaded peptides. The genomic and transcriptomic sequencing data have also been integrated with MS profiling of HLA-associated peptidomes to improve the sensitivity and specificity of neoantigens identification. Neoantigen-based therapies could, however, be quite affordable due to the less expensive high-throughput sequencing and the application of powerful deep learning algorithms.^245,521^ A comprehensive and efficient one-stop computational workflow or a categorized benchmark of the available in silico neoantigen detection approaches is still necessary for clinical application.^522^ The efficient one-stop computational methods might also enable determining the potential of neoantigens as biomarkers for survival prognosis or ICB response prediction using huge data cohorts. Most crucially, the accuracy of these epitope prediction pipelines should also be confirmed by thorough immunomonitoring in early clinical investigations to improve the development of neoantigen-based cancer therapies. In addition to these in silico approaches that predict the immunogenic neoantigens and cognate TCRs based on high-throughput sequencing data, several T cell antigen discovery strategies have recently been developed to unbiased map the immunogenic neoantigens. A variety of pMHCs libraries, including yeast display library, SABRs, BATTLES, have been established, which allow for the flexible and scalable screening of antigenic epitopes. By relying on the physiological activity of T cell killing rather than evaluating TCR-pMHC binding affinity, T-Scan enables the interrogation of a significantly larger antigen space independent of predictive algorithms. Thus, the simplicity and scalability of T cell ligand discovery techniques will be useful for studying the immunogenicity of candidate neoantigens and aiding the development of novel neoantigen-based immunotherapies.

Off-the-shelf precision immunotherapies against public neoantigens is another possible strategy to overcome the time and financial issues in individualized treatment based on personalized neoantigens.^300^ An excellent public neoantigen shared across patients would be produced by a peptide having a hotspot mutation in a driver gene or a TSG that is presented by a relatively common HLA allele. Public neoantigens are more possibility of being clonally conserved across metastases and systematically reappearing among patients.^523^ Numerous general therapeutic techniques are readily available to target public neoantigens, including vaccines, bsAbs, adoptive transfer of CTLs and TCR-T cells.^300,524^ A library of TCRs that specifically target shared neoantigens in an HLA-specific manner has subsequently been developed for patients with advanced cancers.^446,525–530^ As more public neoantigens and corresponding TCRs are discovered, more patients with frequent genetic alterations that drive cancers will benefit from the public neoantigen-reactive TCR library. In addition, the widespread use of cancer genome sequencing and neoantigen prediction pipelines will facilitate matching patients with therapies that target the public neoantigens of their tumors. Therefore, this off-the-shelf strategy based on public neoantigens is anticipated to shorten the time needed for neoantigen identification and extensive T cell cultures, increasing the application of neoantigen-based therapies in a significant portion of patients.

In addition to the neoantigens generated by spontaneous mutations during carcinogenesis, certain covalent molecules can be employed to induce the production of tumor-specific public neoantigens by modifying hotspot residues in highly recurrent somatic mutations. Covalent KRAS-G12C inhibitors, like ARS1620, irreversibly modify the mutant cysteine. The haptenated peptides that carry a covalently attached small molecule can be presented by MHC-I on the cell surface. The haptenated peptide:MHC complexes can serve as tumor-specific neoantigens, triggering a cytotoxic T cell response.^191,192^ Based on this principle, the mutant tumor suppressor proteins can be targetable by a new class of molecules, which induce neoantigen generation and trigger a specific immune response by covalently modifying the hotspot residues like TP53Y220C and TP53R273C. Therefore, the range of tumor-specific neoantigens suitable for therapeutic targeting can be significantly expanded by haptens that specifically modify a mutant oncoprotein rather than functioning as pharmacologic inhibitors.

The neoantigens provide powerful targets for cancer vaccines, which can not only precisely eliminate residual tumor lesions, but also effectively target distant metastatic cells due to their systemic characteristics. Personalized neoantigen vaccines are produced in accordance with the individual tumor conditions in the following steps: collection of tumor tissues and normal samples, sequencing, and analysis of unique mutations, prediction, and validation of immunogenic neoantigens as well as design and production of vaccines. A variety of platforms, including peptides, nucleic acids, and DCs, can be used to develop vaccines based on predicted personalized or matched public neoantigens. Peptide, RNA and DNA-based neoantigen vaccines are high feasibility, generally safety and economical manufacture. However, the majority of patients have not been reliably induced by peptide-based neoantigen vaccinations to elicit substantial neoantigen-specific CD8^+^ T cell responses. The recent success of the COVID-19 mRNA vaccine has accelerated the development of mRNA-based vaccination for cancers. All the active components of tumor vaccines, such as neoantigens, formulations and delivery systems, have undergone ongoing improvement. Synthetic self-amplifying mRNAs (samRNAs), which contain replicase genes that encode RNA-dependent RNA polymerase (RdRp), are gaining interest due to their higher and longer-lasting expression of antigens compared to conventional mRNA.^531^ The in vivo expression of vaccine neoantigens can also be enhanced by natural carriers of genetic instructions, including adenoviruses (Ads), retroviruses and adeno‐associated viruses (AAV).^532^ In addition, various nanoparticle formations, such as lipid nanoparticles, exosomes, virus-like particles, caged protein nanoparticles, bacterial membrane materials-based nanocarrier, high density lipoprotein-mimicking nanodiscs, polyplexes and polymeric nanoparticles, are being developed to enhance the capacity of transport and tissue penetration, boosting the immunogenicity of personalized vaccinations.^533–539^ Compared to the viral vectors, the nanoparticles can also efficiently co-delivery vaccines and immune adjuvants to lymphoid organs, strengthening the neoantigen presentation.

Ex vivo loading of blood-isolated monocytes or hematopoietic progenitor cells with tumor neoantigens effectively improves the anti-cancer effects of neoantigen-based vaccines. Autologous DCs can be loaded the neoantigens in the forms of peptides, RNA and DNA. Compared to the time-and cost-intensive sequencing and computational analysis of patient-specific neoantigens, using autologous WTLs is a more convenient and economical method to induce neoantigen-specific immune responses. Whole tumor cells have both MANA and non-mutated TAAs, which might overcome the potential immune escape and resistance.^367–369^ However, the higher abundance of nonimmunogenic self-antigens might limit the capacity of neoantigens to elicit immune responses. Various immunosuppressive factors are also present in WTLs, which inhibit DC maturation and T cell activation.^30,540^ To overcome these issues, extracellular vesicles (EVs) produced by tumor cells have recently been shown to be a vaccination platform that supports DC maturation and neoantigen presentation. Tumor cell-derived EVs can deliver tumor antigen repertoires into DCs and facilitate neoantigen cross-presentation. EVs also have high immunostimulatory factors that trigger DCs to release innate immune signals.^541–543^ In addition to tumor cell-derived EVs, DC-derived EVs can also serve as a neoantigen-presenting unit to immune cells.^544,545^ Though modulating the tumor immune microenvironment and systemic immune responses, EV-based vaccination can turn a ‘cold’ tumor into a ‘hot’ one. Therefore, EVs may provide an option format for neoantigen-based cancer vaccines, which may potentially be given orally.

Although neoantigen-based immunotherapies have shown promising outcomes in earlier preclinical and clinical investigations, significant advancements are still required, especially for patients with epithelial malignancies. Cancer cells have evolved inherent defenses to evade immune recognition at every stage of the cancer-immunity cycle.^7,461^ Given a complicated mechanisms of immune escape in cancers, combination therapies that simultaneously target different stages of the cancer-immunity cycle may be more effective (Fig. 6). The neoantigen generation and release will be boosted by the cell death following chemotherapy, radiation, targeted therapy, photodynamic therapy and oncolytic viral therapies, which further enhance the anti-tumor immunity cycle. Neoantigen presentation can be facilitated by the administration of IFN-α, GM-CSF, anti-CD40, TLR agonist and STING agonists.^546–553^ To promote the infiltration of immune cells into tumors, the TME modification method and intratumor cytokines can be used.^554,555^ The ICBs and IDO inhibitor will also alter the immunosuppressive TME to enhance neoantigen-based immunotherapy.^556,557^ Nano- and EV-based drug delivery systems have been recently employed as an integrated platform for simultaneous administration of numerous drugs or therapeutic medications that work in concert to activate different stages of cancer-immunity cycle, reverse the immunosuppression and create an immunosupportive TME.^558–560^ These combining strategies using therapeutic agents with different mechanisms of action induce a robust effective, long-lasting and tumor-specific immune response in cancer patients.
